# Age‐Dependent Histone Deacetylase 3 Regulation by βA3/A1‐Crystallin and Inositol Hexaphosphate in Retinal Pigmented Epithelial Cells Reveals a Novel Pathway in Age‐Related Macular Degeneration

**DOI:** 10.1111/acel.70163

**Published:** 2025-07-15

**Authors:** Sujan Chatterjee, Sayan Ghosh, Zachary Sin, Vishnu Suresh Babu, Loretta Viera Preval, Emily Davis, Nguyen Tran, Sridhar Bammidi, Pooja Gautam, Stacey Hose, Yuri Sergeev, Miguel Flores‐Bellver, Kevin Ritter, Henning J. Jessen, Issam Al Diri, Debasish Sinha, Prasun Guha

**Affiliations:** ^1^ Nevada Institute of Personalized Medicine University of Nevada Las Vegas Nevada USA; ^2^ The Wilmer Eye Institute The Johns Hopkins University School of Medicine Baltimore Maryland USA; ^3^ Department of Ophthalmology University of Pittsburgh School of Medicine Pittsburgh Pennsylvania USA; ^4^ National eye Institute National Institutes of Health Bethesda Maryland USA; ^5^ CellSight ‐ Ocular Stem Cell and Regeneration Program, Department of Ophthalmology University of Colorado School of Medicine Aurora Colorado USA; ^6^ Faculty of Chemistry and Pharmacy, Institute of Organic Chemistry University of Freiburg Freiburg Germany

**Keywords:** atrophic age‐related macular degeneration, casein kinase II (CK2), endoplasmic reticulum stress, HDAC3, histone acetylation, inositol hexaphosphate, inositol polyphosphate multikinase, βA3/A1‐crystallin

## Abstract

Age‐related macular degeneration (AMD), a leading cause of vision loss affecting retinal pigment epithelial (RPE) cells, remains largely unexplained by current genome‐wide association studies (GWAS) risk variants. Our research on *Cryba1*, encoding βA3/A1‐crystallin protein, reveals its crucial role in RPE cell function via a novel epigenetic mechanism, also evident in human atrophic AMD samples. Loss of *Cryba1* in mouse RPE cells triggers epigenetic changes by reducing histone deacetylase 3 (HDAC3) activity through two mechanisms. First, *Cryba1* depletion reduces inositol polyphosphate multikinase (IPMK) expression, which potentially reduces inositol hexakisphosphate (InsP6) generation since IPMK's kinase activity is essential for producing InsP4 and InsP5 as precursors to InsP6. Since InsP4, InsP5, or InsP6 is crucial for HDAC3's interaction with the corepressor's DAD domains, reduced IPMK expression in *Cryba1*‐depleted cells likely diminishes the HDAC3‐DAD interaction, leading to a reduction in HDAC3's activity. Second, reduced βA3/A1 protein in *Cryba1*‐deficient cells impairs HDAC3's interaction with casein kinase 2 (CK2), resulting in decreased HDAC3 phosphorylation. Collectively, this increases H3K27 acetylation at the RET promoter region, likely enhancing the transcription of RET, a receptor tyrosine kinase critical for cell survival. Although RET is transcriptionally increased, *Cryba1* loss disrupts its protein maturation, causing immature RET protein accumulation. This triggers age‐dependent endoplasmic reticulum (ER) stress, potentially contributing to the pathogenesis of AMD. Interestingly, although *Cryba1* is not identified as an AMD‐linked variant in current GWAS, its loss may be linked to AMD mechanisms. These findings underscore the potential of gene‐agnostic and epigenetic therapeutic strategies for treating AMD.

## Graphical abstract

βA3/A1‐crystallin regulates HDAC3 activity and epigenetic processes in the RPE. (A) The illustration demonstrates that βA3/A1‐crystallin plays a crucial role in maintaining IPMK protein expression, which in turn activates HDAC3. (B) When βA3/A1‐crystallin is absent (*Cryba1 KO* ), IPMK protein expression decreases, resulting in the deactivation of HDAC3. This deactivation leads to increased H3K27 acetylation at the RET promoter, subsequently enhancing RET gene transcription. In knockout cells (*Cryba1* ^KO^) lacking βA3/A1‐crystallin, RET protein maturation is impaired, causing immature RET to accumulate within the cell. This intracellular sequestration of immature RET ultimately triggers endoplasmic reticulum stress.

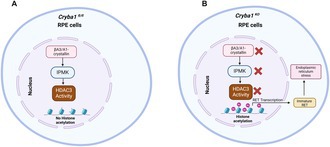

## 1 Introduction

The retinal pigment epithelial (RPE) cells serve as specialized phagocytes of the eye and persevere throughout an organism's entire lifespan (Boya et al. 2023). These post‐mitotic cells play a vital role in maintaining the homeostasis of the neural retina, a function essential for normal vision (Boya et al. 2023). In recognizing the pivotal role of RPE in ocular health, it has become evident that alterations in RPE function can trigger the atrophic (dry) form of age‐related macular degeneration (AMD) (Boya et al. 2023; Ghosh et al. 2018; Handa et al. 2019; Shang et al. 2021; Valapala et al. 2014; Wang et al. 2018), a debilitating eye disease prevalent in the aging population with limited therapeutic options. Despite Genome‐Wide Association Studies (GWAS) identifying several AMD risk variants, their effects remain modest, leaving approximately 70% of AMD risk unexplained. Notably, early‐stage AMD is associated with decreased chromatin accessibility in the RPE (Wang et al. 2018), suggesting a potential link between epigenetic changes in RPE cells and disease onset. Additionally, it has been previously shown that in age‐related multifactorial neurodegenerative diseases like Alzheimer's, Parkinson's disease, and in particular AMD, epigenetic changes can play a critical role in disease pathogenesis (Mallik et al. 2022; Sharma et al. 2020; Song et al. 2023). In particular, class I, II, and III HDACs such as HDAC3, HDAC4/5, and Sirtuin 1(Sirt1), respectively, have been shown to have critical roles in transcription repression during aging and neurodegenerative diseases (Morris and Monteggia 2013; Razick et al. 2023). However, they differ significantly in structure and mechanism of deacetylation. The enzymatic activity of most class I HDACs such as HDAC3 relies on recruitment into multi‐subunit corepressor complexes like the SMRT co‐repressor's deacetylase activation domain (DAD) and association with inositol tetraphosphate (Ins(1,4,5,6)P4), which are brought to chromatin by repressive transcription factors, potentially explaining the regulatory role of inositol phosphates in transcription (Watson et al. 2012). Inositol phosphates have also been associated with aging mechanisms involving the immune system and the brain cortex (Samochocki and Strosznajder 1995; Xu et al. 2013). Furthermore, the enzymatic activity of most Class II HDACs depends on their interaction with the N‐CoR, BCoR, MEF2 transcription factor, and CtBP corepressors (Bertos et al. 2001). Their activity is also regulated through subcellular compartmentalization controlled by site‐specific phosphorylation and binding of 14‐3‐3 proteins (Bertos et al. 2001). Additionally, Class III HDACs such as Sirt1 are dependent on NAD+ and are controlled by a C‐terminal regulatory element (CTR) where the catalytic domain‐CTR interface brought about by the interaction between β‐hairpin structure of the CTR and the β‐sheet of the NAD+ ‐binding domain regulates the enzyme activity (Davenport et al. 2014). These findings highlight the notion that, in a multifactorial aging disease like AMD, epigenetic factors like HDACs may exert a substantial influence beyond genetic variations.

AMD remains a significant challenge despite two decades of GWASs identifying over 60 risk loci (He et al. 2024). The lack of effective treatments for atrophic AMD highlights the need to explore novel targets and signaling pathways, particularly epigenetic changes affecting RPE health and disease progression. Our research has revealed the critical role of crystallins in RPE function, with particular focus on βA3/A1‐crystallin encoded by the *Cryba1* gene (Boya et al. 2023). While crystallins were initially characterized as lens‐specific proteins, where they constitute over 90% of total soluble protein and create the transparency essential for vision, they also represent an interesting example of “gene sharing” or protein moonlighting, having been recruited from pre‐existing proteins with diverse functions (Zigler and Sinha 2015). The β/γ‐crystallin superfamily exhibits a distinctive structure, since each polypeptide contains two domains composed of two “Greek key” motifs (four‐stranded anti‐parallel beta sheets). What makes βA3/A1‐crystallin unique is that the *Cryba1* gene produces two polypeptides through leaky ribosomal scanning, βA3‐crystallin (215 residues) and βA1‐crystallin (198 residues). These isoforms are identical except for 17 additional amino acids at βA3's N terminus, suggesting they may be directed to different subcellular compartments with distinct functions. This structural diversity underlies βA3/A1‐crystallin's functional versatility across tissues. In the lens, it primarily serves as a structural protein, whereas in RPE cells and astrocytes, it performs various regulatory functions critical for maintaining cellular and tissue homeostasis (Zigler and Sinha 2015). Mechanistically, βA3/A1‐crystallin exhibits distinct subcellular localizations and functions depending on cell type. In astrocytes, it is found in both nucleus and cytoplasm, where it regulates Notch signaling, astrocyte template formation, and retinal vascular patterning (Zigler and Sinha 2015). In RPE cells, however, it localizes to the lysosomal lumen, interacting with the V_0_ subunit of V‐ATPase, a proton pump essential for lysosomal acidification, thereby regulating mTORC1 signaling, phagocytosis, and autophagy (Valapala et al. 2014). βA3/A1‐crystallin also modulates phosphoinositide metabolism through binding to PITPβ, influencing PI(4,5)P2 levels, ezrin phosphorylation, amino acid regulation, inflammation, microvilli organization, and EGFR signaling (Shang et al. 2021). These processes are crucial for maintaining RPE polarity and preventing epithelial–mesenchymal transition (EMT), with abnormalities in these pathways implicated in AMD. Our previous studies with RPE‐specific knockout mice (*Cryba1* cKO) have demonstrated that loss of βA3/A1‐crystallin leads to an age‐dependent atrophic AMD‐like phenotype characterized by drusen‐like deposits, lipofuscin accumulation (autofluorescence), large vacuoles/debris, melanosome accumulation in the RPE, inflammatory changes, photoreceptor loss, and declining retinal function (Ghosh et al. 2018; Shang et al. 2021; Zigler and Sinha 2015).

Given the limited availability of human atrophic AMD tissue, mouse models provide crucial platforms for studying the underlying mechanisms of multifactorial diseases such as AMD. While mice lack the distinct anatomical structures of nonhuman primates or human eyes, particularly the fovea/macula, functional studies have revealed important similarities. The central mouse retina shares functional characteristics with the peripheral human macula, especially regarding photoreceptor density and phagocytic load of the RPE (Volland et al. 2015). This is significant because macular degeneration often begins in these peripheral macular regions (Volland et al. 2015). Therefore, mouse models such as the *Cryba1* cKO serve as valuable tools for investigating the complex mechanisms underlying AMD, including epigenetic changes in the RPE. Our research with this RPE‐specific knockout model offers critical insights into how epigenetic alterations contribute to AMD development, providing a foundation for validating these findings in human disease contexts. The involvement of RPE‐dependent changes in AMD pathogenesis is now well‐established (Ghosh et al. 2018; Shang et al. 2021; Zigler and Sinha 2015), aligning with our findings on βA3/A1‐crystallin's crucial role in RPE function. While the initial insult, whether aging, genetics, environmental factors, oxidative stress, or inflammation, and the precise location of disease onset remain uncertain, the RPE's central role is increasingly evident. Although various studies indicate that photoreceptors, Bruch's membrane, and the choriocapillaris also contribute to AMD development (Boya et al. 2023), the RPE's strategic anatomical position and its diverse functions in maintaining photoreceptor health and retinal homeostasis highlight its significance (Ghosh et al. 2018). Recent evidence further supports this premise, showing that global chromatin accessibility decreases in the RPE of dry/atrophic AMD eyes, while similar changes in the neurosensory retina occur only in advanced disease stages (Wang et al. 2018). This temporal sequence of epigenetic alterations provides compelling evidence that RPE dysfunction represents a critical and potentially initiating event in AMD progression, reinforcing the relevance of our *Cryba1* cKO model for understanding the molecular mechanisms of this complex disease.

Our study reveals a novel regulatory axis in RPE cells where βA3/A1‐crystallin orchestrates epigenetic regulation through dual mechanisms: facilitating HDAC3‐CK2 interaction for phosphorylation and maintaining optimal InsP6 levels by restoring IPMK protein level critical for HDAC3 function (Watson et al. 2016). This previously unrecognized role of βA3/A1‐crystallin in epigenetic regulation provides fundamental insights into RPE cell homeostasis and suggests that targeting the βA3/A1‐crystallin‐HDAC3 axis, particularly through modulation of InsP6 signaling or HDAC3 activity, could offer therapeutic opportunities for treating non‐exudative AMD. Furthermore, our findings underscore the importance of investigating non‐GWAS‐identified genes in complex diseases like AMD, where understanding the interplay between genetic and epigenetic factors may be crucial for developing effective treatments.

## 2 Results

### 2.1 Loss of βA3/A1‐Crystallin in the RPE Triggers Reduction in HDAC3 Activity and Altered Epigenetic Signatures

To understand how βA3/A1‐crystallin regulates epigenetic factors in the RPE, we first analyzed the RNA‐seq data from RPE cells of *Cryba1* cKO mice (Ghosh et al. 2019). Our analysis revealed that the loss of βA3/A1‐crystallin has a significant impact on global transcription, leading to the upregulation of 144 genes and the downregulation of 114 genes (Figure S1A, Tables S1 and S2). Histone acetylation is a reversible epigenetic modification crucial in regulating gene expression (Allis and Jenuwein 2016). This dynamic process involves the addition of acetyl groups to histone proteins, which can directly influence the accessibility of DNA and, consequently, the activation or repression of specific genes (Allis and Jenuwein 2016). Interestingly, we observed a marked increase in global H3/H4 acetylation in *Cryba1* cKO RPE cells compared to *Cryba1* floxed (*Cryba1* ^fl/fl^) controls, including H3K9, H3K18, H3K27, H4K12, and H4K16 acetylation (Figure S2A–J). These effects closely resembled the impact of suberoylanilide hydroxamic acid (SAHA; Figure S2J), a pan‐histone deacetylase inhibitor used as a positive control, which is known for enhancing global histone acetylation (Allis and Jenuwein 2016).

**FIGURE 1:**
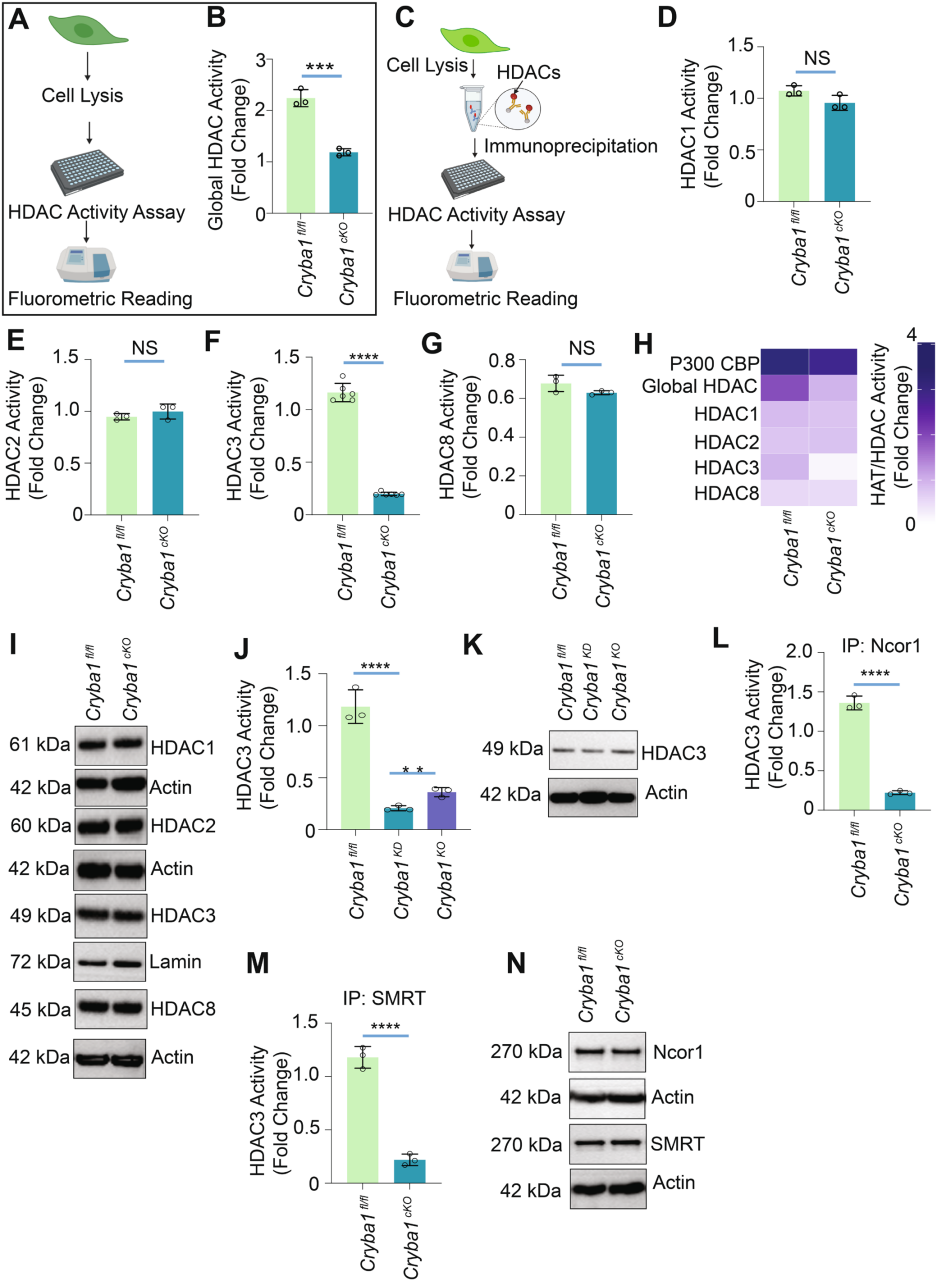
*Cryba1* Deletion diminishes HDAC3 activity in mouse RPE cells. (A) Schematic diagram of global HDAC activity assay. Nuclear lysate was isolated followed by measuring HDAC activity fluorometrically in a 96‐well dark half area opaque microtiter plate. BOC‐(Ac)‐Lyc‐AMC was used as a substrate. (B) The lysate was isolated from *Cryba1* ^fl/fl^ and *Cryba1* ^cKO^ mouse RPE cells, followed by a class I HDAC activity assay. Deletion of *Cryba1* in the cKO RPE diminished the enzymatic activity of Class I HDAC. The result was represented as fold change compared with blank value and summerize of three individual experiments (*n* = 3, ***p < 0.001). (C) Schematic depicting immunoprecipitation of selective HDAC followed by activity assay. HDAC1 (D), HDAC2 (E), HDAC3 (F), and HDAC8 (G) were immunoprecipitated from *Cryba1* ^fl/fl^ and *Cryba1* ^cKO^ mouse RPE cells followed by evaluation of activity assay for the respective HDACs. HDAC3 activity was decreased in *Cryba1* ^cKO^ compared to *Cryba1* ^fl/fl^ (F). The activity of HDAC1/2 and 8 in *Cryba1* ^cKO^ was comparable to *Cryba1* ^fl/fl^. IgG was used as a negative control. Data has been presented as fold change compared with IgG. Results are summerize of three individual experiments (*n* = 3, ****p < 0.0001, NS = not significant). (H) Heatmap of enzymatic activity of all class I HDACs (Histone deacetylase) and histone acetylase p300. Data has been presented as fold change compared with IgG. (I) Immunoblot analysis of HDAC1, HDAC2, HDAC3, and HDAC8 from the whole cell lysate isolated from *Cryba1* ^fl/fl^ and *Cryba1* ^cKO^ mouse RPE cells demonstrate comparable amounts of protein levels in the RPE cells from both genotypes. Actin (cytosolic) and lamin (nuclear) were used as loading controls. The result was of three individual experiments. (*n* = 3). (J) HDAC3 was immunoprecipitated from *Cryba1* ^fl/fl^, βA1 KD (Knock Down) and βA3 KO (Knockout) RPE cells, followed by a HDAC3 activity assay. HDAC3 activity significantly decreased in both βA1^KD^ and βA3^KO^ cells, while βA1^KD^ showed a greater reduction in HDAC3 activity than βA3^KO^ cells. IgG was used as negative control. Data has been presented as fold change compared with IgG value. (*n* = 3, ****p < 0.0001 vs. βA1 KD, **p < 0.01 vs. βA3 KO). (K) Immunoblot analysis of HDAC3 from the total RPE lysate isolated from *Cryba1* ^fl/fl^, βA1 KD and βA3 KO. The result was of three individual experiments. (*n* = 3). (L) NCor1, which is an HDAC3 complex protein, was immunoprecipitated from *Cryba1* ^fl/fl^ and *Cryba1* ^cKO^ RPE cell lysates followed by HDAC3 activity. The data demonstrates a marked loss of HDAC3's enzymatic activity in *Cryba1* ^cKO^ compared to *Cryba1* ^fl/fl^. IgG was used as a negative control. Data has been presented as fold change compared with IgG value. The result was summerize of three individual experiments (*n* = 3, ****p < 0.0001). (M) SMRT, which is another HDAC3 complex protein, was immunoprecipitated from *Cryba1* ^fl/fl^ and *Cryba1* ^cKO^ RPE cell lysates followed by HDAC3 activity. The data demonstrates loss of enzymatic activity of HDAC3 in *Cryba1* ^cKO^ compared to *Cryba1* ^fl/fl^. IgG was used as a negative control. Data has been presented as fold change compared with IgG value. The result was of three individual experiments (*n* = 3, ****p < 0.0001). (N) Immunoblot analysis of NCor1 and SMRT from the total lysates isolated from *Cryba1* ^fl/fl^ and *Cryba1* ^cKO^ RPE cells demonstrating comparable protein levels in both lysates, while actin was used as a loading control. The result was of three individual experiments (*n* = 3).

In cells, histone acetylation levels are controlled by histone “writers” such as P300 and CBP, which add acetyl groups to histone lysines, and “erasers” such as histone deacetylases (HDACs), which remove acetyl groups from histone lysines (Dancy and Cole 2015). Our hypothesis suggests two possibilities: (i) the loss of *Cryba1* increases the activity of histone acetyltransferase (HAT), or (ii) it reduces the activity of HDACs, resulting in a significant rise in histone acetylation. Interestingly, when *Cryba1* is deleted, global class I HDAC activity is notably reduced in the cKO RPE, compared to floxed controls (Figure 1A,B), but HAT activity remains unchanged (Figure S3A). A comprehensive examination of all class I HDACs (HDAC1, 2, 3, and 8) revealed that the loss of *Cryba1* selectively diminished HDAC3 activity (> 82.97%), leaving other class I HDACs unaffected (Figure 1C–H). Intriguingly, *Cryba1* loss did not alter the levels of HDAC3 protein (Figure 1I). Moreover, reintroducing *Cryba1* using an adenoviral vector in cKO RPE cells restored HDAC3 activity to near‐normal levels, unlike the control vector (Figure S3B). Additionally, *Cryba1* overexpression reduced histone acetylation levels, particularly for H3K9 and H3K27 (Figure S3C–F). These findings highlight the role of *Cryba1* in selectively regulating HDAC3 activity and modulating histone acetylation in RPE cells.

*Cryba1* produces two protein isoforms, βA3‐ and βA1‐crystallin, from the same mRNA by leaky ribosomal scanning (Ghosh et al. 2019). It is now recognized that different isoforms resulting from alternative translation pathways may exhibit unique and novel functionalities (Ghosh et al. 2019). Notably, our findings revealed that RPE cells obtained from CRISPR/cas9 gene‐edited mice with βA1‐crystallin knockdown (KD) had a more pronounced decrease in HDAC3 activity than those with βA3‐crystallin knockout (KO); however, the protein levels of HDAC3 remained unchanged in both mouse lines (Figure 1J,K).

It is known that HDAC3 requires direct binding to silencing mediator of retinoic acid and thyroid hormone receptor (SMRT) or nuclear receptor corepressor 1 (NCoR1) corepressor proteins to function as a deacetylase in cells (Watson et al. 2012, 2016). In our investigation into HDAC3 regulation, we conducted immunoprecipitation of both NCoR1‐HDAC3 and SMRT‐HDAC3 corepressor complexes from *Cryba1* cKO and floxed RPE cells. The data demonstrated a substantial reduction (> 83.7% and 81.4%) in HDAC3 activity within both complexes in *Cryba1* cKO RPE cells (Figure 1L,M) despite no changes in the protein levels for both NCoR1 and SMRT (Figure 1N). This suggests that βA3/A1‐crystallin likely modulates HDAC3 activity in RPE cells independently of corepressor complexes.

### 2.2 Loss of βA3/A1‐Crystallin Causes Abnormal Acetylation Signatures in the RET Gene and Contributes to ER Stress in RPE Cells

**FIGURE 2:**
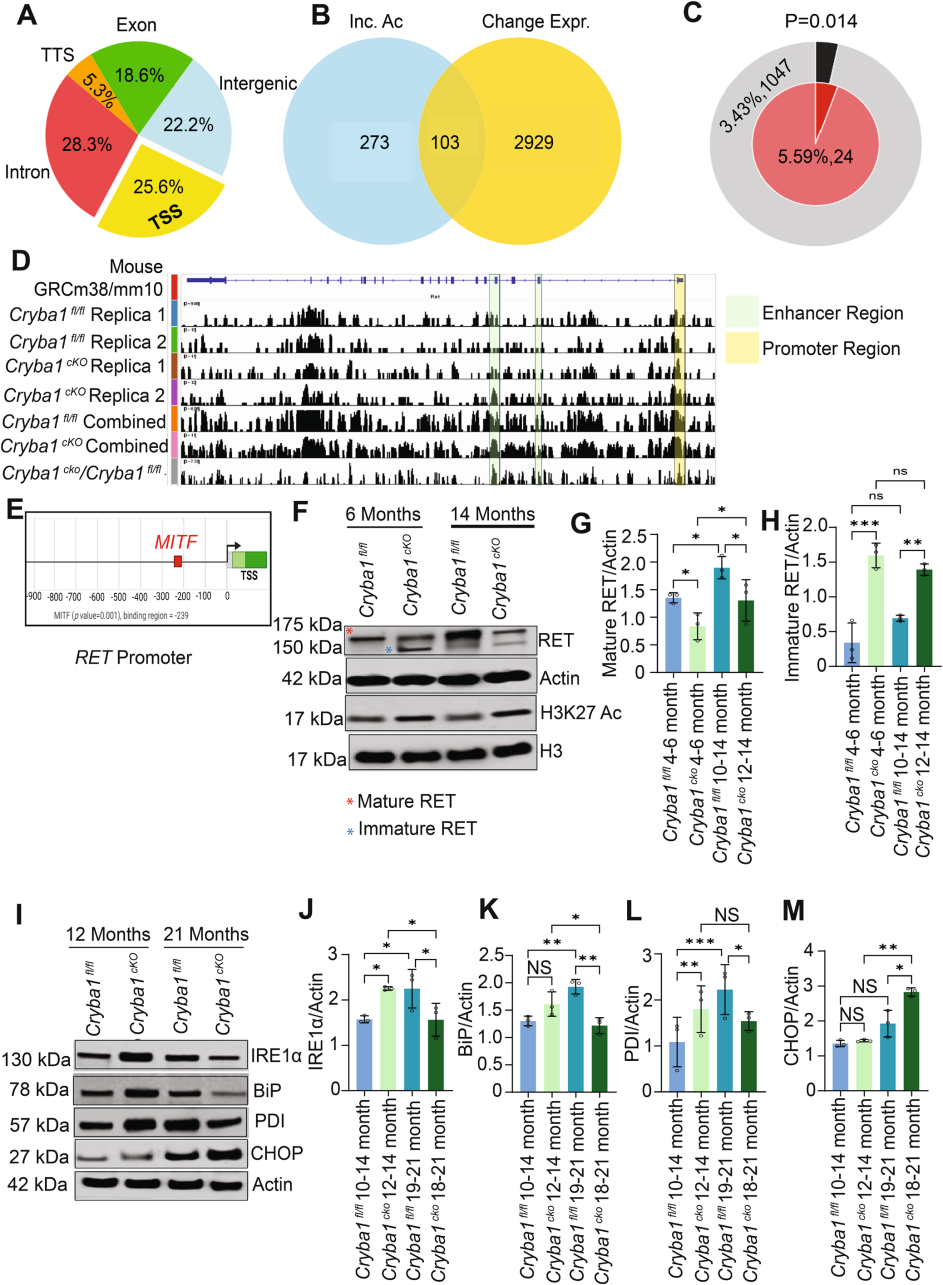
Transcriptionally enriched regions on the RET gene provide a cue for altered ER regulation in *Cryba1* cKO RPE cells. (A) Distribution of significant hyperacetylated peaks across different genomic regulatory regions. (B) The overlap between genes linked to hyperacetylated peaks in genomic regulatory regions and those exhibiting an upregulation in expression based on our RNA sequencing data. (C) Pathway enrichment analysis revealed that differentially expressed genes are enriched for pathways associated with endoplasmic reticulum stress (*p* = 0.014). The outer pie (gray/black) shows the percentage of genes in the background gene list that are associated with endoplasmic reticulum stress. The inner pie (red) shows the percentage of differentially expressed genes that are associated with endoplasmic reticulum stress. The whole genome was used as the background gene list. (D) ChIP‐seq analysis was performed using Cryba1fl/fl (*n* = 2) and Cryba1cKO (*n* = 2) mouse RPE samples. Multiple genomic regions of the RET gene showing differential acetylation peaks were analyzed using DESeq2 (*p* ≤ 0.05, FC ≥ 1.5) and visualized using the Integrated Genomics Viewer (IGV) aligned to the GRCm38/mm10 mouse reference genome. Each column represents a replicate, along with the combined analysis of differential acetylation peaks identified in Cryba1fl/fl and Cryba1cKO samples. Green rows indicate significant peaks identified in the enhancer region, while yellow rows indicate significant peaks in the promoter region of RET. (E) MITF transcription factor binding sites in the RET promoter region were identified and predicted using the Eukaryotic Promoter Database (EPD) (*p* ≤ 0.001). MITF binds 239 bp upstream of the RET gene's transcription start site (TSS). (F) Western blot showing mature and immature RET protein and H3K27Ac protein expression in 6‐month‐old wild‐type (Cryba1fl/fl) and Cryba1cKO mice, and 14‐month‐old Cryba1fl/fl and Cryba1cKO mice. Actin was used as a loading control. Densitometry analysis showing (G) mature RET protein levels (H) immature RET protein levels in 4–6 and 10–14 month‐old Cryba1fl/fl and Cryba1cKO mice. *n* = 3 biological replicates. The data points passed the Shapiro–Wilk normality test and statistical analysis was performed using two‐way ANOVA followed by Tukey's post hoc test. *p ≤ 0.05, **p ≤ 0.01, and ***p ≤ 0.001. (I) Western blot showing protein levels of endoplasmic reticulum (ER) stress markers in 10–14 month and 18–21‐month‐old Cryba1fl/fl and Cryba1cKO mice. Actin was used as a loading control. Densitometry analysis showing (J) IRE1α protein levels (K) BiP protein levels (L) PDI protein levels and (M) CHOP protein levels in 10–14 month and 18–21‐month‐old Cryba1fl/fl and Cryba1cKO mice. *n* = 3 biological replicates. The data points passed the Shapiro–Wilk normality test and statistical analysis was performed using two‐way ANOVA followed by Tukey's post hoc test. *p ≤ 0.05, **p ≤ 0.01, and ***p ≤ 0.001.

Chromatin immunoprecipitation sequencing (ChIP‐seq) analysis of acetylated H3K27 from *Cryba1* cKO RPE cells revealed significant alterations in acetylation patterns across various genomic regulatory regions influencing biological pathways including stress response and apoptotic pathways (Figure S4A). Our investigation focused on hyperacetylated peaks, which indicate areas of open chromatin potentially facilitating gene expression. We observed a notable distribution of these hyperacetylated peaks across different genomic regulatory regions in the *Cryba1* cKO RPE cells (Figure 2A). To elucidate the relationship between hyperacetylation and changes in gene expression, we conducted an overlap analysis between the RNA sequencing data showing increased expression in *Cryba1* cKO RPE cells (Figure S1A) and the regions exhibiting increased acetylation (Table S3, Extended Excel 3). This revealed a noticeable intersection between genes associated with hyperacetylated peaks in genomic regulatory regions and genes showing a significant increase in expression from our RNA sequencing data (Figure 2B,C). Of particular interest are increases in acetylation at transcription start site (TSS), as these could expose DNA to transcription factors, potentially increasing gene expression. Our analysis identified RET (proto‐oncogene tyrosine‐protein kinase receptor) as having both a significant increase in TSS acetylation (Figure 2D) and a marked upregulation in gene expression (Figure S1), probably owing to the MITF (a major transcription factor in the RPE cells) binding site at the RET promoter (Ma et al. 2019; Figure 2D,E). This finding was subsequently validated through western blot analysis. Interestingly, our data revealed a significant accumulation of immature RET protein (150 kDa) and a corresponding reduction in mature RET protein (175 kDa) levels in the RPE cells from *Cryba1* KO mice (Figure 2F–H). RET, a receptor tyrosine kinase crucial for cell survival, is typically located in the cell membrane (Guo et al. 2023). The glycosylation of RET protein, which increases its molecular weight (175 kDa), is essential for its maturation and membrane localization (Guo et al. 2023). These alterations in the RET protein profile in *Cryba1* KO RPE cells suggest an impairment in the proper maturation and trafficking of this protein, potentially leading to compromised RPE cell survival, functionality, and retinal homeostasis, which are key aspects in the pathophysiology of AMD (Zigler and Sinha 2015). This observation suggests an accumulation of unprocessed RET within the cell, which could potentially trigger endoplasmic reticulum (ER) stress, as when RET remains in its immature form (150 kDa), it may not undergo proper glycosylation or cleavage, which can lead to its accumulation in the ER and induce stress responses (Jing et al. 2012).

To explore the possibility of ER stress response in the RPE cell, we performed western blot analysis for ER stress responsive proteins such as IRE1α/β, BiP, PD1, and CHOP (Chen et al. 2023) in WT and *Cryba1* KO RPE. Our results revealed increased expression of IRE1α/β and PD1 in the RPE cells from younger *Cryba1* KO mice, but a decline in aged animals (Figure 2I–L). Interestingly, CHOP, which has been implicated in programmed cell death in response to impaired function of the ER (Chen et al. 2023) showed significant increase in aged *Cryba1* KO RPE cells with no noticeable change in its expression in young animals (Figure 2I,M). This differential expression of ER stress response regulators such as IRE1α/β, PD1, and CHOP in *Cryba1* KO RPE cells suggests a shift toward cell death from the cellular response to ER stress with increasing age. The decline of IRE1α/β and PD1 in aged compared to young RPE cells implies a diminished adaptive response to ER stress over time, potentially accelerating cell damage. Furthermore, increased expression of CHOP in aged *Cryba1* KO RPE cells indicates a potential progression toward ER stress‐induced apoptosis (Chen et al. 2023), which may contribute to cellular dysfunction and degeneration in the absence of *Cryba1*. While we identified additional genes exhibiting enrichment of acetylated H3K27 in cKO mice (Table S3, Extended Excel 3), those potentially involved in AMD or age‐related diseases will be investigated in subsequent studies.

### 2.3 βA3/A1‐Crystallin Regulates HDAC3 Phosphorylation/Activity by Modulating Its Binding With CK2

To elucidate the mechanisms through which βA3/A1‐crystallin influences the activity of HDAC3 in RPE cells, we utilized the UCSF Chimera program for protein modeling and observed the potential formation of a complex involving βA3/A1‐crystallin (depicted in light blue), HDAC3 (depicted in orange), and the deacetylase‐activation domain (DAD) from the SMRT corepressor (depicted in magenta) as illustrated in (Figure S5A).

To gain further insight into these protein–protein interactions, co‐immunoprecipitation studies on RPE cells from *Cryba1*‐floxed mice, which overexpress either RFP (adenovirus‐RFP infected) or RFP‐βA3/A1‐crystallin (adenovirus‐RFP‐*Cryba1*), were performed. These studies utilized anti‐RFP beads, followed by immunoblotting for HDAC3 and RFP proteins, demonstrating a robust binding between βA3/A1‐crystallin and HDAC3 (Figure S5B). This observation was supported by copurification experiments using endogenous proteins (βA3/A1‐crystallin and HDAC3) and HDAC3 pulldown followed by immunoblot for both proteins from *Cryba1*‐floxed RPE cells, indicating a binding interaction. However, such an interaction was not observed when the proteins were copurified from *Cryba1* cKO RPE cells (Figure S5C). To further validate the physical interaction between HDAC3 and βA3/A1‐crystallin, an in vitro pull‐down experiment was also conducted. Equal concentrations of recombinant *Cryba1*‐myc were incubated with recombinant HDAC3‐GST and immunoprecipitated with GST followed by immunoblot, which revealed binding between the two recombinant proteins (Figure S5D). These results confirm the physical interaction between βA3/A1‐crystallin and HDAC3. To determine the subcellular localization of the *Cryba1*‐HDAC3 interaction, we employed a proximity ligation assay (PLA) followed by confocal microscopy analysis, which revealed that endogenous βA3/A1‐crystallin and HDAC3 proteins interact predominantly within the nucleus (Figure S5E).

**FIGURE 3:**
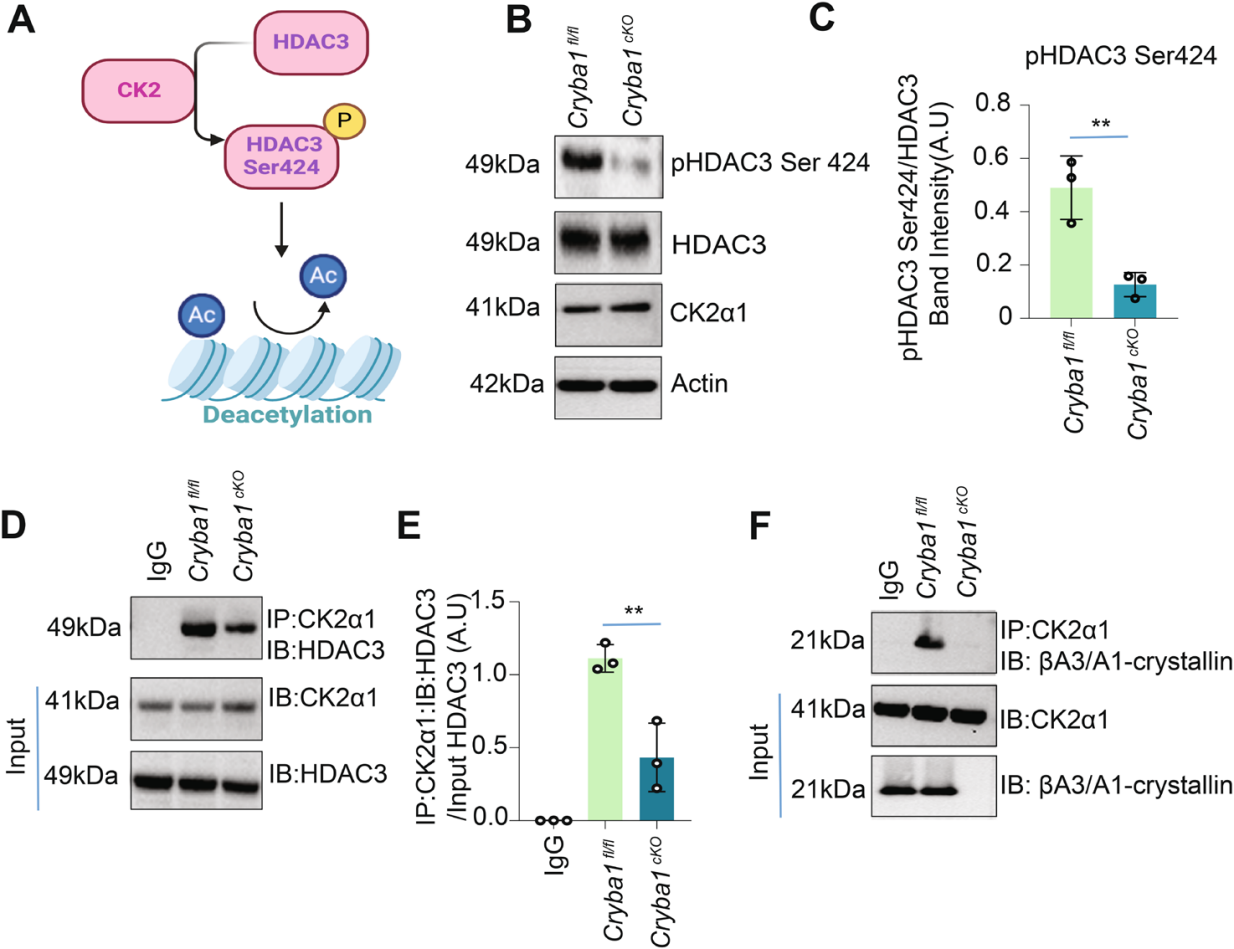
Cryba1 regulates HDAC3 phosphorylation. (A) Pictorial representation demonstrates that CK2‐mediated HDAC3 phosphorylation is essential for activating HDAC3's deacetylase activity. (B) Immunoblot analysis shows that Cryba1 deletion reduced HDAC3 phosphorylation at Serine 424. Actin was used as a loading control. The result was of three individual experiments (*n* = 3). (C) Densitometric analysis showing decreased phospho‐HDAC3‐Ser424 phosphorylation in Cryba1cKO compared to Cryba1fl/fl (*n* = 3, **p < 0.01). (D) Endogenous immunoprecipitation study depicted that Cryba1 deletion diminished endogenous CK2α1 binding to HDAC3. The result was of three individual experiments. (*n* = 3). (E) Densitometric analysis confirmed that *Cryba1* deletion diminished endogenous CK2α1 binding to HDAC3. The result was of three individual experiments (*n* = 3, ***p < 0.001). (F) Endogenous CK2α1 binds to *Cryba1* when immunopurified from *Cryba1* ^fl/fl^ RPE cells but not *Cryba1* ^cKO^. The result was of three individual experiments. (*n* = 3).

Next, we wondered how *Cryba1* binding to HDAC3 influences the activation of HDAC3's deacetylase activity. It has been shown that phosphorylation of HDAC3 at serine 424 by CK2 is crucial in activating the enzymatic function of HDAC3 (Zhang et al. 2005; Figure 3A). Our data showed approximately a 60% decrease in HDAC3 phosphorylation upon deletion of *Cryba1* in the RPE cells (Figure 3B,C). Remarkably, this reduction was effectively reversed upon overexpression of *Cryba1* (βA3/A1‐crystallin adenoviral vector) in the *Cryba1* cKO RPE cells (Figure S6A,B). Considering the potential interaction between βA3/A1‐crystallin and HDAC3 indicated by protein modeling and pull‐down assays, we investigated whether the deletion of *Cryba1* could affect the interaction between CK2 and HDAC3, consequently impacting CK2‐mediated HDAC3 phosphorylation. Our data from endogenous pull‐down assays demonstrated a marked reduction in the interaction between CK2 and HDAC3 upon *Cryba1* deletion (Figure 3D,E). This suggests that βA3/A1‐crystallin may serve as a linker protein, facilitating interaction between CK2 and HDAC3. To substantiate this premise, we performed additional endogenous pull‐down assays confirming the direct interaction between βA3/A1‐crystallin and CK2 (Figure 3F). These results suggest that βA3/A1‐crystallin may play a crucial role as a linker protein, mediating the interaction between CK2 and HDAC3, thereby influencing HDAC3 phosphorylation and enzymatic activity.

### 2.4 βA3/A1‐Crystallin Depletion in the RPE Cells Alters the InsP6‐Mediated Regulation of SMRT (NcoR2) and NCoR1‐Dependent HDAC3 Activity

**FIGURE 4:**
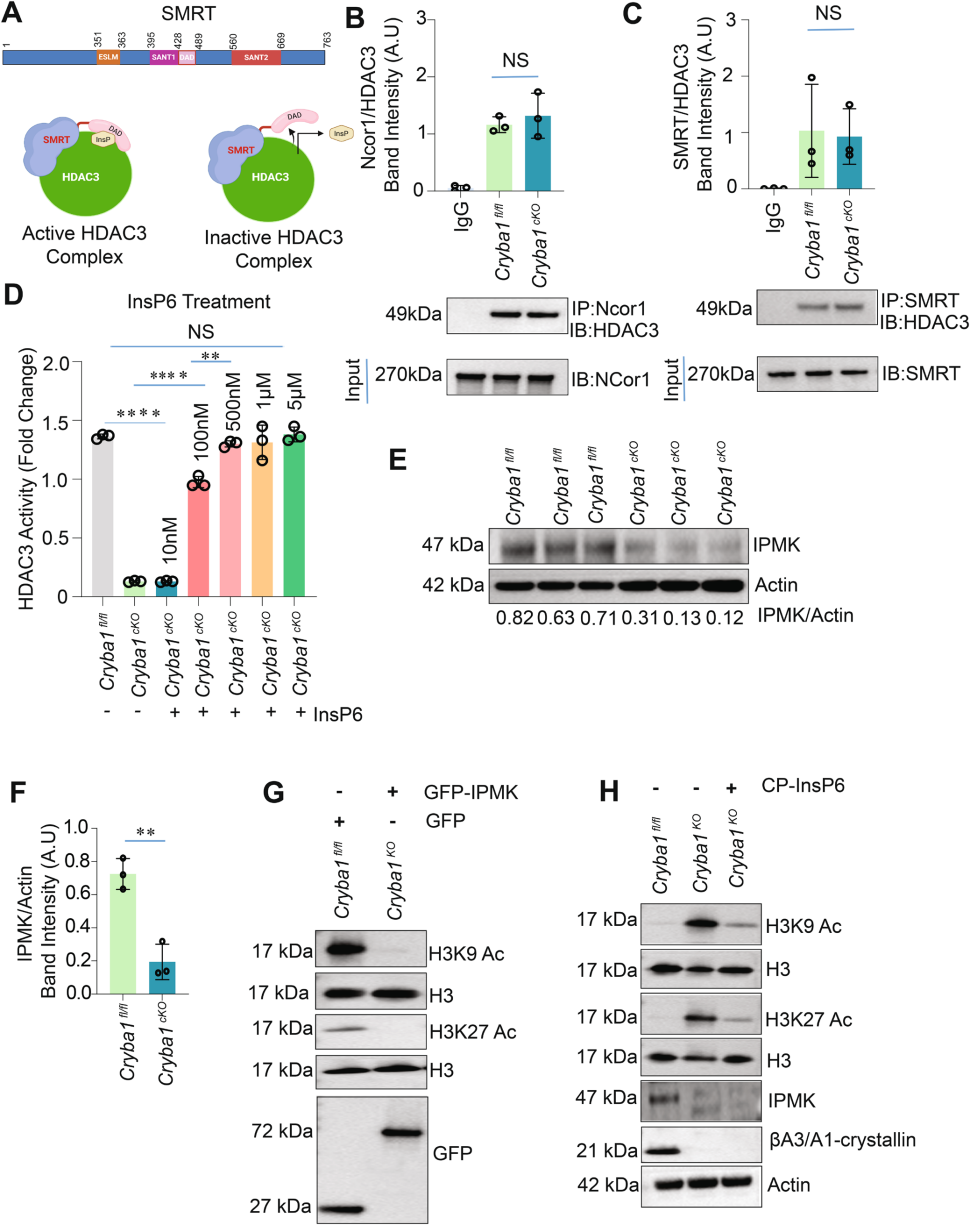
IP6 is essential for HDAC3 activity. (A) Pictorial representation of SMRT protein domains, which includes the SANT1, SANT2, ESLM (ELM2 specific motif), and DAD domains. The model represents that inositol phosphate (InsP) is required for the DAD domain of SMRT protein to interact with HDAC3. (B) An endogenous immunoprecipitation study determined that interaction of HDAC3 and Ncor1 in *Cryba1* ^cKO^ cells was comparable to *Cryba1* ^fl/fl^. The HDAC3/NCor1 complex was pulled down by immunoprecipitating NCor1 followed by western blot for HDAC3. IgG was used as a negative control, and the immunoblot of NCOR1 from the cell lysate was used as an input control. The western blot data is also represented by densitometric analysis. The result was of three individual experiments (*n* = 3, NS = not significant). (C) The interaction of HDAC3 and SMRT in *Cryba1* ^cKO^ cells was comparable to *Cryba1* ^fl/fl^. HDAC3 complex integrity with corepressor SMRT was analyzed through immunoprecipitation of SMRT from *Cryba1* ^fl/fl^ and *Cryba1* ^cKO^ RPE cell lysates using anti‐SMRT antibody followed by immunoblotting for HDAC3. Immunoblotting against anti‐SMRT antibodies from respective cell lysates was used as input control, while IgG was used as a negative control. The data is also represented by densitometric analysis. The result was of three individual experiments. (*n* = 3, NS = not significant). (D) InsP6 increased HDAC3 activity in a dose‐dependent manner in vitro when purified from *Cryba1* ^cKO^ cells. To analyze the role of inositol phosphate in HDAC3 activity, endogenous HDAC3 was immunopurified from *Cryba1* ^fl/fl^ and *Cryba1* ^cKO^ RPE nuclear lysates, followed by dose‐dependent IP6 treatment in vitro. (*n* = 3, **p < 0.01, ***p < 0.001, ****p < 0.0001). (E, F) Immunoblot analysis shows that *Cryba1* deletion diminished IPMK at the protein level in RPE cells from *Cryba1* ^cKO^ compared to *Cryba1* ^fl/fl^. Actin was used as a loading control. The result was of three individual experiments. (*n* = 3, ***p < 0.001). (G) *Cryba1* Knock out cells overexpressed with GFP‐control and IPMK‐WT‐GFP virus. Results demonstrated that GFP‐control (Empty GFP) *was* unable to rescue H3K9 and H3K27 acetylation as compared with IPMK‐Wild Type (GFP‐IPMK)‐GFP. Immunoblot analysis against an anti‐GFP antibody confirmed the efficacy of transfection. Results are of two individual experiments. (*n* = 2). (H) cell‐permeable InsP6 (CP‐InsP6) treatment at a dose of 50 μM for 24 h successfully rescued H3K9, H3K27 acetylation as compared with untreated *Cryba1* ^fl/fl^ cells. Lysates were immunoblotted against acetylated anti‐H3K9, anti‐H3K27 antibodies, followed by stripping and reprobing against anti‐H3 antibody respectively. Lysates were immunoblotted again anti‐IPMK, anti‐CrybA1 and anti‐ action antibodies, used as loading control. Data represents two experimental replicates (*n* = 2).

HDAC3's enzymatic activity is critically dependent on its binding to corepressor proteins SMRT or NCOR1, with the DAD domain playing a crucial role in this interaction (Watson et al. 2012, 2016; Figure 4A). Recent crystal structure and biochemical analysis has revealed that a highly charged higher order inositol phosphate (InsP 4, 5, or 6) coordinates the HDAC3‐DAD interaction (Watson et al. 2012, 2016). The loss of βA3/A1‐crystallin, which diminishes HDAC3 activity (Figure 1F), does not disrupt the integrity of the HDAC3‐corepressor complex (Figure 4B,C), suggesting a potential loss of DAD domain interaction (Watson et al. 2012, 2016). Notably, HDAC3 purified from *Cryba1* cKO RPE cells restored activity when treated with InsP6 in vitro, in a dose‐dependent manner (Figure 4D). At 100 nM, more than 50% of the activity was rescued, while 500 nM restored activity to levels comparable to the *Cryba1* ^fl/fl^ control. This rescue data suggest that the loss of βA3/A1‐crystallin may impede the higher order inositol phosphate (HOIP) pathway, diminishing InsP levels. IPMK (inositol polyphosphate multikinase), the rate‐limiting enzyme in the HOIP pathway (Chatterjee et al. 2024; Guha et al. 2019), was found to be substantially reduced in RPE cells lacking βA3/A1‐crystallin (Figure 4E,F). Overexpression of wild‐type IPMK in *Cryba1* cKO RPE cells restored histone acetylation levels by reducing H3K9 and H3K27 acetylation (Figure 4G), strongly suggesting that the loss of βA3/A1‐crystallin reduces IPMK levels, subsequently impairing HDAC3 activation due to reduced intracellular InsP levels. To further confirm this hypothesis, *Cryba1* cKO cells were treated with cell‐permeable InsP6. Regular InsP6 (phytic acid) is impermeable to cells due to its high negative charge, so a chemically modified form was used (Kim et al. 2019). This modified form masks the charged phosphates, allowing cellular entry, and the mask is dismantled once inside the cells, releasing active InsP6 (Pavlovic et al. 2015). Treatment with cell‐permeable InsP6 reduced H3K9/27 acetylation in cKO RPE cells to levels comparable to wild‐type (*Cryba1* ^fl/fl^) RPE cells, while IPMK expression remained reduced (Figure 4H). This confirms that the loss of βA3/A1‐crystallin reduces HDAC3 activity and elevates histone acetylation by reducing IPMK protein expression, which can be rescued by InsP6 treatment.

### 2.5 Diminished HDAC3 Activity in RPE Cells of Human Atrophic AMD Donors Was Associated With Increased Histone Acetylation

**FIGURE 5:**
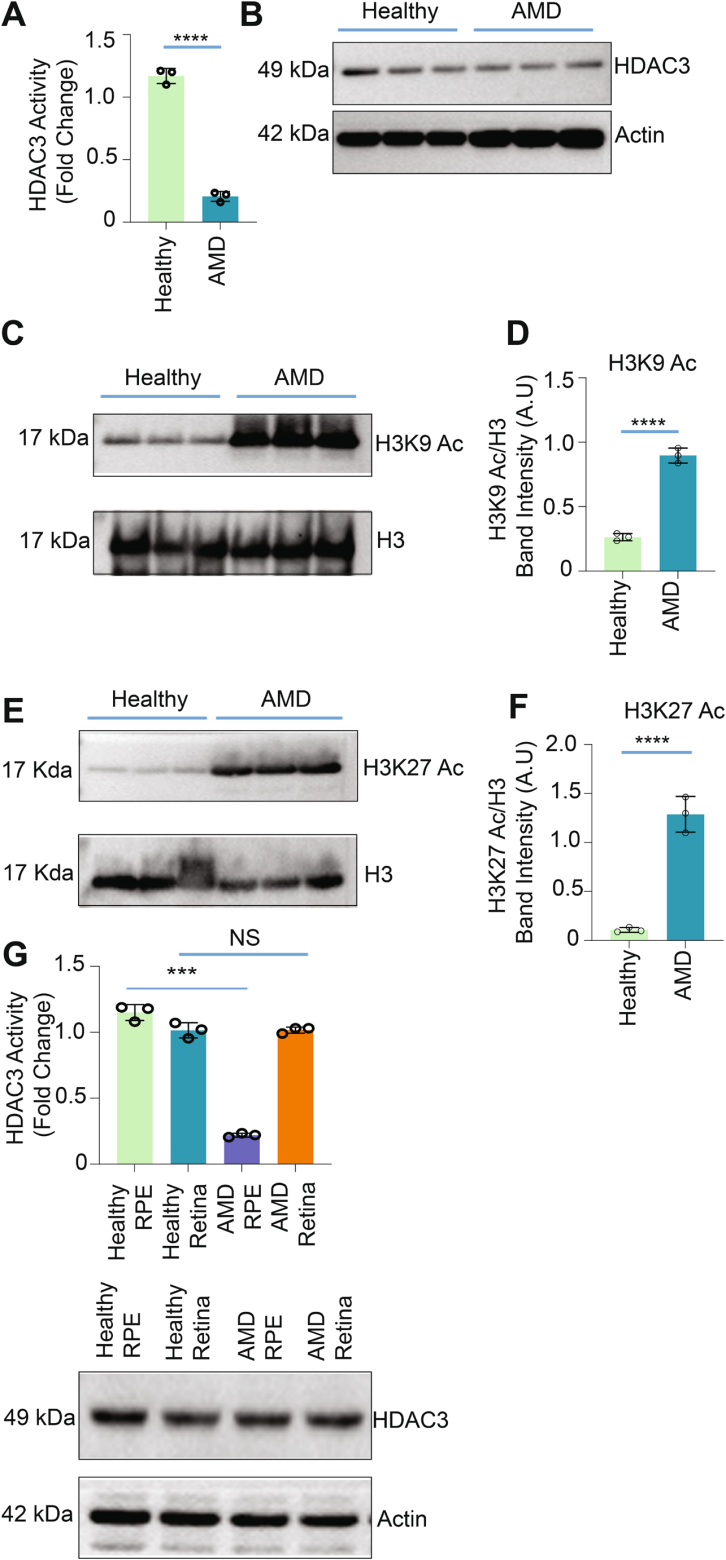
HDAC3 activity is diminished in AMD patients. (A) HDAC3 activity was diminished in AMD patients compared with healthy human individuals. HDAC3 was immunopurified from RPE cells of healthy and AMD patients, followed by a HDAC3 activity assay. Results are of three independent experiments. (*n* = 3, ****p < 0.0001). (B) The HDAC3 protein level in AMD patients was comparable to that of healthy human individuals. Actin was used as a loading control. Results are of three independent experiments (*n* = 3). (C–F) Western blot analysis of H3K9 and H3K27 acetylation (C, E) from healthy and AMD patient RPE cells showing a marked increase in H3k9/27 acetylation in AMD RPE cell lysates when compared with healthy individuals. Respective immunoblots were stripped and then reprobed with an anti‐H3 antibody, which was used as a loading control. Results are of three independent experiments. (D, F) Represent densitometric analysis of H3k9/27 acetylation. *n* = 3 (****p < 0.0001, ***p < 0.001). (G) HDAC3 activity was exclusively diminished in the RPE cells but not in the retina of AMD patients. HDAC3 was immunopurified from RPE cells and retina of healthy patients and those suffering from AMD, followed by a HDAC3 activity assay. The HDAC3 protein level in AMD patients was comparable to healthy individuals. Actin was used as a loading control. Results are of three independent experiments. (*n* = 3, ****p < 0.0001).

Since we observed marked alterations in histone acetylation and HDAC3 activity in RPE cells from *Cryba1* cKO mice, which exhibit an atrophic AMD‐like phenotype, we next investigated the status of these epigenetic mediators in individuals with the atrophic form of AMD. Our data showed that in the RPE of human AMD donor eyes graded by the Minnesota Grading System for disease severity (Olsen et al. 2017), HDAC3 activity was significantly reduced (Figure 5A) relative to age‐matched controls, without concurrent changes in HDAC3 protein levels (Figure 5B). In addition, there was a significant increase in the acetylation levels of H3K9 (Figures 5C,D and S7A,B) and H3K27 (Figures 5E,F and S7C,D) in RPE cells from individuals with AMD. Interestingly, HDAC3 activity was not significantly different in normal and AMD retina samples from the same donors (Figure 5G). Hence, our study suggests that decreased HDAC3 activity and increased histone acetylation specifically in RPE cells may play a crucial role in AMD, and understanding the underlying mechanism could lead to a novel treatment strategy for this condition.

## 3 Discussion

Epigenetic changes refer to heritable or reversible alterations in gene expression or phenotype through mechanisms such as DNA methylation and histone modifications, which are influenced by environmental factors without involving changes to the DNA sequence (Allis and Jenuwein 2016). These epigenetic alterations within chromatin play a fundamental role in regulating various biological processes, and their dysregulation has been linked to aging hallmarks and the development of complex age‐related diseases, including cancer, Alzheimer's, and Huntington's diseases (Mallik et al. 2022; Morris and Monteggia 2013). HDAC3 has been implicated in these diseases through its involvement in chromatin remodeling and gene expression regulation (Zhang et al. 2005). Additionally, HDAC3‐mediated repression of transcription has been associated with cognitive decline both in aging and neurodegenerative diseases (He et al. 2023). However, the role of Class I HDACs, specifically HDAC3, in the pathogenesis of atrophic AMD has not been previously explored. In this study, we show a significant reduction in HDAC3 activity both in AMD human patients (Figure 5A) and in a mouse AMD model (Figure 1F). Interestingly, while HDAC inhibitors have demonstrated therapeutic potential in treating neurodegenerative diseases like Alzheimer's (He et al. 2023), our results suggest that an alternative therapeutic strategy may be required for AMD. Our findings indicate that, rather than inhibiting HDAC3, enhancing its activity in RPE cells may be a more effective therapeutic approach for AMD, owing to the reduction in HDAC3 activity rather than expression in both human and mouse RPE cells during AMD progression. Furthermore, we showed that βA3/A1‐crystallin, a moonlighting protein with diverse cellular functions, regulates HDAC3 activity in RPE cells. Our study indicates that βA3/A1‐crystallin interacts with HDAC3 to influence its activity (Figure S5A–E), highlighting an unexpected role of crystallin in regulating chromatin accessibility and transcription through epigenetic modifications. Previous research has shown that HDAC3 also influences major transcription regulators such as HIF‐1α (Wu et al. 2011) and Yin Yang 1 (YY1) (Sankar et al. 2008), which play critical roles in RPE/photoreceptor health and disease (Forooghian et al. 2007; Fu et al. 2021). This suggests a strong correlation between HDAC3 loss in cKO mice or AMD patients and potential downstream transcriptional changes in RPE cells during AMD.

Furthermore, we observed that the loss of βA3/A1‐crystallin leads to a reduction in HDAC3 activity, and this deficit can be rescued by the addition of InsP6 (Figure 4D,E). InsP6, a naturally occurring compound found in foods like rice, potatoes, and legumes (Pujol et al. 2023), has been implicated in cellular processes such as chromatin remodeling and gene expression (Gonzalez et al. 2010). Our results suggest that InsP6 may activate HDAC3 in RPE cells by modulating the complex formation between HDAC3 and its DAD domain.

Our study also uncovered a crucial role for CK2‐mediated HDAC3 phosphorylation in regulating its enzymatic activity (Zhang et al. 2005). Loss of *Cryba1* in RPE cells triggered impairment of this phosphorylation (Figure 3B,C), and we identified βA3/A1‐crystallin as a linker protein necessary for the HDAC3‐CK2 interaction (Figure 3D–F).

Interestingly, InsP6 can bind to CK2 and enhance its kinase activity (Lee et al. 2013). In cKO mice, the significant reduction in IPMK protein levels (Figure 4E), along with decreased CK2‐mediated HDAC3 phosphorylation (Figure 3B,C), suggests that reduced InsP6 production may lower CK2 activity and further diminish HDAC3 phosphorylation. Taken together, our results suggest that potentially reduced InsP6 levels resulting from the loss of IPMK in *Cryba1* cKO RPE cells may impair HDAC3 activation through two mechanisms: (i) disruption of the DAD‐HDAC3 complex and (ii) partial inhibition of CK2 kinase. These observations open up a new avenue for therapeutic intervention in AMD by targeting HDAC3 regulation and restoring its activity through either βA3/A1‐crystallin overexpression or InsP6 supplementation.

At the downstream transcriptional level, our study reveals a novel mechanism linking βA3/A1‐crystallin deficiency to RPE cell dysfunction through epigenetic regulation and ER stress pathways. We demonstrated that loss of βA3/A1‐crystallin leads to reduced HDAC3 activity, resulting in hyperacetylation of chromatin, particularly at the RET gene promoter (Figure 2D). This increased acetylation may facilitate MITF binding (Figure 2E) and subsequent expression of RET, a receptor tyrosine kinase crucial for cell survival, typically located in the cell membrane (Guo et al. 2023). The glycosylation of RET protein, which increases its molecular weight, is essential for its membrane localization (Guo et al. 2023). Our research revealed an accumulation of immature RET (Figure 2F–H), coinciding with increased transcription (Figure S1A). This observation supports the hypothesis that in *Cryba1* KO mice, the loss of membrane‐bound RET protein in RPE cells triggers downstream epigenetic changes, leading to enhanced transcription. However, due to defective glycosylation, this results in the accumulation of immature RET. Importantly, the buildup of unprocessed RET appears to induce an age‐dependent ER stress response in RPE cells (Figure 2I–M). In young *Cryba1* KO mice, we observed elevated levels of adaptive ER stress markers (IRE1α/β and PD1), indicating an initial compensatory response (Figure 2I–M). However, this protective mechanism appears to fail with age, as evidenced by the decline in IRE1α/β and PD1 levels in aged KO RPE cells, coupled with a significant increase in the pro‐apoptotic factor CHOP (Figure 2I–M). This age‐dependent shift from adaptive to pro‐apoptotic ER stress response suggests that chronic ER stress, initially triggered by impaired protein processing, may be a key mechanism driving RPE cell dysfunction and degeneration in the absence of βA3/A1‐crystallin.

**FIGURE 6:**
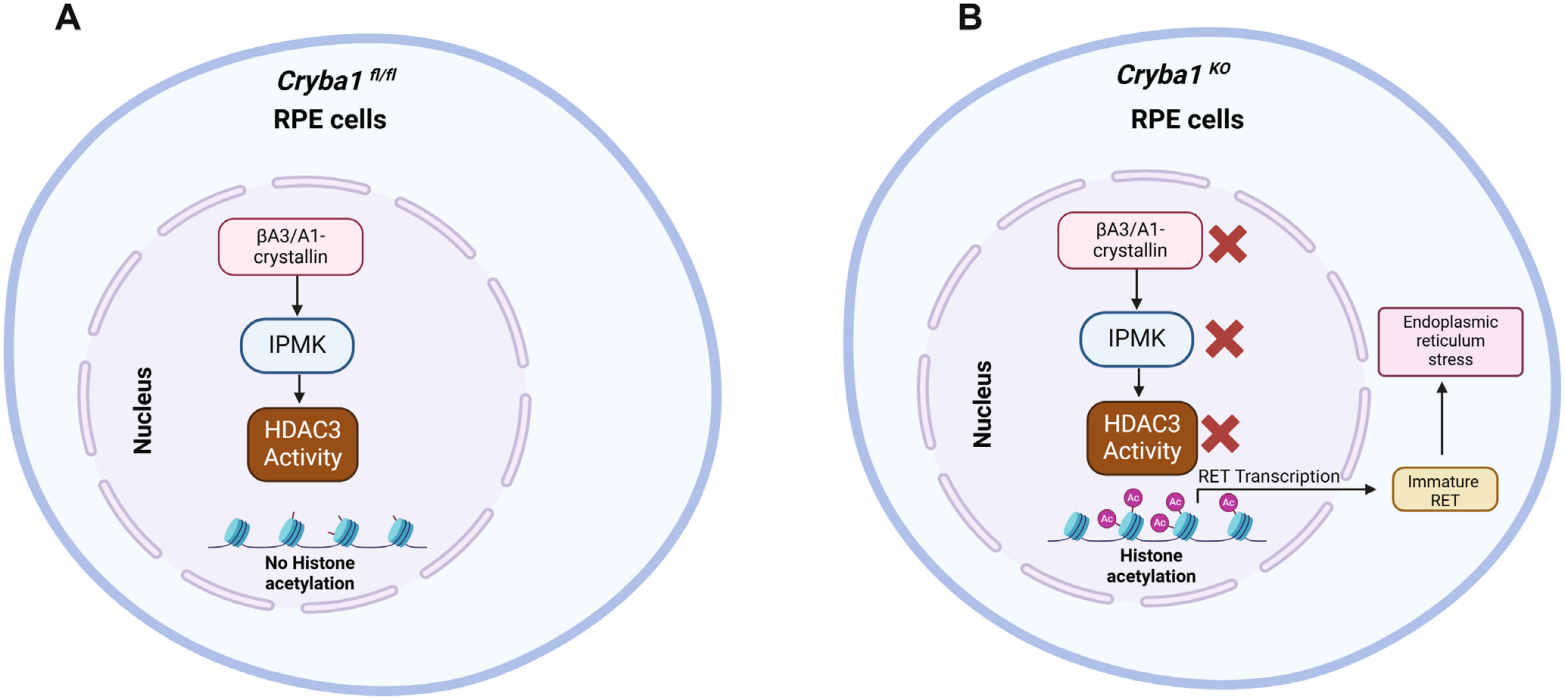
βA3/A1‐crystallin regulates HDAC3 activity and epigenetic processes in the RPE. (A) The illustration demonstrates that βA3/A1‐crystallin plays a crucial role in maintaining IPMK protein expression, which in turn activates HDAC3. (B) When βA3/A1‐crystallin is absent, IPMK protein expression decreases, resulting in the deactivation of HDAC3. This deactivation leads to increased H3K27 acetylation at the RET promoter, subsequently enhancing RET gene transcription. In knockout cells (*Cryba1* KO) lacking βA3/A1‐crystallin, RET protein maturation is impaired, causing immature RET to accumulate within the cell. This intracellular sequestration of immature RET ultimately triggers endoplasmic reticulum stress.

Our findings reveal a complex interplay between βA3/A1‐crystallin, HDAC3 activity, and ER stress in RPE cells, establishing a previously unrecognized mechanistic link in AMD pathogenesis (Figure 6). The identification of βA3/A1‐crystallin as a crucial regulator of HDAC3 activity through both CK2‐mediated phosphorylation and InsP6‐dependent complex formation provides new insights into the epigenetic regulation of RPE cell function. Furthermore, the age‐dependent transition from adaptive to maladaptive ER stress responses in *Cryba1*‐deficient RPE cells suggests a temporal component in disease progression that may explain why AMD primarily affects aging populations. These discoveries not only advance our understanding of AMD pathobiology but also present multiple therapeutic opportunities. Potentially, InsP6 supplementation or enhancement of βA3/A1‐crystallin function could restore HDAC3 activity and normalize chromatin accessibility, while interventions mitigating ER stress might help maintain RPE cell viability. As AMD continues to be a leading cause of vision loss worldwide (Kaarniranta et al. 2023; Zigler and Sinha 2015), these novel therapeutic approaches could provide much‐needed alternatives to current treatments, particularly for patients with atrophic AMD, where treatment options remain limited. Future studies investigating the clinical applicability of these findings may lead to the development of more effective treatments for this devastating disease.

## 4 Materials and Methods

### 4.1 Animals

All animal studies were conducted in accordance with the Guide for the Care and Use of Animals (National Academy Press) and were approved by the University of Pittsburgh Animal Care and Use Committee. Both male and female mice were used in this study. RPE‐specific βA3/A1‐crystallin conditional (*Cryba1* cKO), as well as global βA3 KO and βA1 KD mice were generated on a C57BL/6J background as previously described. All mice that were used in this study were negative for the RD8 mutation. Animals were euthanized as per approved guidelines, and the RPE was harvested for downstream experiments (immunoblot, HDAC activity assays) as explained previously (Ghosh et al. 2018; Shang et al. 2021; Valapala et al. 2014).

### 4.2 RPE Explant Culture and Pan‐HDAC Inhibitor SAHA Treatment as Well as Adenovirus‐*Cryba1* Construct Infection

Eyes from 4 to 5 month‐old *Cryba1*‐floxed and cKO mice were enucleated, and the RPE‐choroid‐sclera (RCS) complex was harvested and placed onto PVDF membranes after flattening them by making several relaxing cuts. The explants were then cultured face up in complete media as previously described. *Cryba1* ^fl/fl^ RPE explants were incubated with 2 μM of SAHA (Sigma Aldrich, Cat# SML0061‐5MG) and incubated overnight, followed by estimation of histone acetylation by immunoblot. *Cryba1* cKO RPE explants were also infected with either adenovirus(Ad)‐*Cryba1* construct or vehicle control at a dose of 10^5^ vg/mL for 24 h, and subsequent experiments were performed (Shang et al. 2021).

### 4.3 Human AMD Donors

The human AMD donor sections were provided by Dr. Flores‐Bellver's laboratory at the University of Colorado. The donors were all of Caucasian descent and had an average age of 78 ± 3 years. The control donors did not have any eye diseases. Both sexes were included in the study, and *n* = 6 individual donors were included for both control and AMD. The disease was staged according to the 4‐point Minnesota Grading System (MGS), and all the controls were classified as MGS1 (no AMD) and the AMD donors were classified as MGS2 (early AMD; Olsen et al. 2017).

### 4.4 Western Blot Analysis

Cell lysates were prepared through syringe flush by using a salt‐free (NaCl‐free) lysis buffer (50 mM Tris pH 7.5, 50 mM potassium acetate, 5% v/v glycerol, 0.3% v/v Triton X‐100, one tablet of Roche complete protease inhibitor). We used salt‐free (NaCl‐free) lysis buffer for all the assays because a high a salt concentration dissociates higher‐order inositol phosphates (HOIPs) from HDAC3/1, which is essential for their deacetylase activity. Samples were centrifuged at 15,000 g for 10 min, and the protein concentration of the supernatant was measured. Proteins were resolved by SDS‐polyacrylamide gel electrophoresis (NuPAGE Bis‐Tris Midi GEL, Life Technologies, Cat #: WG1402BX10) and transferred to Immobilion‐P PVDF (Millipore‐Sigma, Cat #: IPVH00010) transfer membranes. The membranes were incubated with primary antibody diluted in 3% BSA in Tris‐buffered saline with Tween 20 (20 mM Tris–HCl, pH 7.4, 150 mM NaCl, and 0.02% Tween 20) overnight at 4°C. Respective antibodies for western blot were anti‐Cryba1 (Abcam, Cat #: ab151722), anti‐HDAC3 (Santa Cruz Biotechnology, Cat #: sc‐376957), anti‐HDAC1 (Santa Cruz Biotechnology, Cat #: sc‐81598), anti‐HDAC2 (Santa Cruz Biotechnology, Cat #: SC‐7899), anti‐HDAC8 (Biolegend, Cat #: 685504), anti‐pHDAC3 Ser424 (Thermo Fischer Scientific, Cat #: PA5‐99339), anti‐CK2 (ProteinTech, Cat #: 10992‐1‐AP), anti‐β‐actin (ProteinTech, Cat #: 81115‐1‐RR), anti‐lamin (ProteinTech, Cat #: 12987‐1‐AP), anti‐SMRT (Bethyl Laboratories, Cat #: A301‐148A), anti‐Ncor1 (Cell Signaling Technology, Cat #: 5948), anti‐H3 (Cell Signaling Technology, Cat #s: 12648 and 4499), anti‐H3K8Ac (Cell Signaling Technology, Cat #: 2598), anti‐H3K9Ac (Cell Signaling Technology, Cat #: 9649), anti‐H3k18Ac (Cell Signaling Technology, Cat #: 13998), anti‐H3K27Ac (Abcam, Cat #: ab4729; Invitrogen, Cat#: PA5‐40096), anti‐H3K56Ac (Cell Signaling Technology, Cat #: 4243), anti‐H4K8Ac (Cell Signaling Technology, Cat #: 2594), anti‐H4K12Ac (Cell Signaling Technology, Cat #: 13944), anti‐H4K16Ac (EMD Millipore Corp., Cat #: 07‐329) and anti‐Myc (ProteinTech, Cat #:16286‐1‐AP), RET (Cell Signaling Technology, Cat #: 14556) and ER Stress Sampler Kit (Cell Signaling Technology, Cat #: 9956), particularly antibodies against BiP, IRE1α, PDI, CHOP, and β‐actin (loading control, Cell Signaling Technology, Cat #: 4970S). Following primary antibody incubation, the PVDF membrane was washed three times with Tris‐buffered saline/Tween‐20, and incubated with HRP‐conjugated secondary antibody (ECL, Cat #: NA934V and NA931V), and the bands visualized by chemiluminescence (Super Signal West Pico, Pierce, Cat #: 34579). The depicted blots are representative replicates selected from at least three experiments. Densitometric analysis was performed using ImageJ software. Blots for acetylated histones were stripped using Restore Western Blot stripping buffer (Thermo Scientific, Cat # 21059), then reprobed with H3 antibody (Cell Signaling Technology, Cat #: 12648S) at room temperature for 2 h followed by secondary antibody incubation and development (Ghosh et al. 2018; Guha et al. 2019).

### 4.5 HAT Activity

HAT activity was measured by following the HAT Assay Kit (Abcam, Cat #: ab‐204709) protocol. *Cryba1* ^fl/fl^ and *Cryba1* cKO cells were lysed in salt‐free (NaCl‐free) lysis buffer (50 mM Tris pH 7.5, 50 mM potassium acetate, 5% v/v glycerol, 0.3% v/v Triton X‐100, Roche complete protease inhibitor). Samples were centrifuged at 15,000 g for 10 min, and the protein concentration of the supernatant was measured. About 20 μg total protein from the lysate was incubated against anti‐p300 CBP HAT antibody (Cell Signaling Technology, Cat #: 86377) at 4°C for 1 h, followed by capturing the antibody with EZview A/G beads (Millipore‐Sigma, Cat #: E3403). Beads were washed three times with an ice‐cold reaction buffer (supplied with kit). Immunoprecipitated P300 was used as a protein source. Absorbance was measured after specific substrate reaction by using a Spectramax iD5 plate reader (Molecular Devices). All measurements were performed in triplicate, and data were analyzed using GraphPad Prism (version 6.0, GraphPad Software Inc).

### 4.6 HDAC Activity

To study global HDAC activity from *Cryba1* ^fl/fl^ and *Cryba1* cKO cell lysates, we used an HDAC activity assay kit (Active Motif, Cat #: 56200). In brief, we lysed cells in a low‐salt lysis buffer, as stated before, followed by centrifugation at 15,000 g to isolate the supernatant. About 20 μg of protein from *Cryba1* ^fl/fl^ and *Cryba1* cKO lysates was incubated with the substrate provided with the kit, and then fluorescence was measured using a plate reader.

To study individual HDAC activity, the HDAC1, 2, 3, and 8 were individually immunopurified, followed by an activity assay using the following kits: HDAC Assay Kit (Active Motif, Cat#: 56200 for HDAC1, 2 and 8) and the HDAC3 Assay Kit (BPS, Cat#: 10186‐628). In brief, *Cryba1* ^fl/fl^ and *Cryba1* cKO cells were lysed in salt‐free (NaCl‐free) lysis buffer (50 mM Tris pH 7.5, 50 mM potassium acetate, 5% v/v glycerol, 0.3% v/v Triton X‐100, one tablet of Roche complete protease inhibitor; Xu et al. 2013). Then, the lysate was centrifuged at 15,000 g for 10 min, and the protein concentration of the supernatant was measured. Around 20 μg total protein from the lysate was incubated against anti‐HDAC1 (Santa Cruz Biotechnology, Cat # sc‐81598), anti‐HDAC2 (Santa Cruz Biotechnology, Cat #: sc‐7899), anti‐HDAC3 (Santa Cruz Biotechnology, Cat #: sc‐81600) and anti‐HDAC8 (Biolegend, Cat #: 685504) antibodies, respectively, at 4°C for 1 h, followed by capturing antibody with EZview A/G beads (Millipore Sigma, Cat #: E3403). Beads were washed 3× with an ice‐cold reaction buffer (supplied with the kit). After a specific substrate reaction, fluorescence was measured using a Spectra max iD5 plate reader (Molecular Devices). All measurements were performed in triplicate, and data was analyzed using GraphPad Prism (version 6.0, GraphPad Software Inc; Watson et al. 2012, 2016).

### 4.7 InsP6 Rescuing HDAC3 Activity

*Cryba1* cKO cells were lysed in salt‐free (NaCl‐free) lysis buffer (50 mM Tris pH 7.5, 50 mM potassium acetate, 5% v/v glycerol, 0.3% v/v Triton X‐100, and one Roche complete protease inhibitor tablet) because high salt concentration dissociates HOIPs from HDAC3/1, which is essential for their deacetylase activity. Samples were centrifuged at 15,000 g for 10 min, and the protein concentration of the supernatant was measured. 20 μg total protein from the lysate was incubated against anti‐HDAC3 antibodies, respectively, at 4°C for 1 h, followed by capturing antibodies with EZview A/G beads (Millipore Sigma, Cat #: E3403). Beads were washed 3× with an ice‐cold reaction buffer (supplied with the kit). Following washing, beads were resuspended in reaction buffer and incubated for 1 h at room temperature with 10, 100, 500 nM, and 1 μM concentrations of IP6 (Sigma Aldrich Cat #: P8810‐100G). After incubation, beads were washed 3× with reaction buffer. HDAC3 activity was measured by the addition of a fluorogenic substrate provided by the HDAC3 Assay Kit (BPS, Cat #: 10186‐628) and read using a Spectramax iD5 plate reader (Molecular Devices). All measurements were performed in triplicate, data analyzed, and the IC50 value was calculated using GraphPad Prism (version 6.0, GraphPad Software Inc; Watson et al. 2012, 2016).

### 4.8 Immunoprecipitation

To ascertain HDAC3 and βA3/A1‐crystallin binding in overexpression conditions, *Cryba1* ^fl/fl^ RPE explants were infected with Ad‐RFP (Vector Biolabs, Cat # 1660) or Ad‐*Cryba1*‐RFP (Vector Biolabs, customized) constructs at 10^5^ vg/mL for 24 h. The RPE lysates were prepared, and immunoprecipitation was performed using anti‐RFP beads, followed by immunoblot for HDAC3. The whole cell lysates were also used to immunoblot for HDAC3 and RFP as input controls. To analyze endogenous binding, RPE cells were lysed in salt‐free lysis buffer (50 mM Tris pH 7.5, 50 mM potassium acetate, 5% v/v glycerol, 0.3% v/v Triton X‐100, and one Roche complete protease inhibitor tablet). For the endogenous immunoprecipitation (IP) study, respective antibodies against βA3/A1‐crystallin, CK2, or HDAC3 were used, followed by western blot of the binding partners. In brief, the IP was performed from 500 μg of protein lysate. Protein lysates were incubated for 2 h at 4°C with respective antibodies; then, the antibody‐protein complex was captured with EZview A/G beads (Millipore Sigma, Cat #: E3403). Beads were pelleted and washed with lysis buffer 3×, followed by elution in sample buffer. Immunoprecipitated samples were resolved on a NuPAGE Bis‐Tris gel, followed by western blotting.

### 4.9 Cell Permeable InsP6 Treatment

Cells plated in a flatmount overnight treated with cell permeable InsP6 (CP‐InsP6) at a dose of 50 μM (Kim et al. 2019; Pavlovic et al. 2015).

### 4.10 In Vitro Binding Assay

Equal amount of recombinant myc‐βA3/A1‐crystallin (Origene, Cat #: TP321965) was co‐incubated with either recombinant GST‐HDAC3 (Signal Chem, Cat #: H85‐30G) or GST (Signal Chem, Cat #: G52‐30U) in lysis buffer, and the complex was maintained for 30 min at 4°C. After the addition of GST beads, incubation continued for an additional 45 min and was washed 3× with ice‐cold lysis buffer. SDS sample buffer was added, and binding was confirmed by western blotting of anti‐Myc antibodies (Guha et al. 2019).

### 4.11 Proximity Ligation Assay (PLA)

Proximity ligation assay was carried out on RPE flat mounts following the manufacturers' protocol (Navinci Diagnostics, NaveniFlex Tissue MR Red, Cat #: NT.MR.100). Antibody for HDAC3 (Cell Signaling Technology, Cat #: 3949) was used at a 1:100 dilution with beta crystallin A3 (Abcam, Cat #: ab‐151722) for the first set of PLA to check the interaction between crystallin and HDAC3.

### 4.12 Molecular Modeling

Models of molecular structure for human βA3/A1‐crystallin, HDAC3 and SMRT‐DAD were obtained from the AlphaFold Protein Structure Database (https://alphafold.ebi.ac.uk/). Molecular modeling of the possible interaction between these proteins was done using the “movement” tool in the extensible molecular modeling system UCSF CHIMERA, VER. 1.17.1 (https://www.cgl.ucsf.edu/chimera/).

### 4.13 RNA‐Seq Data Analysis

The following published RNA‐Seq datasets from 5‐month‐old mouse RPE cells were downloaded: GSM4043956 (*Cryba1* ^fl/fl^ (WT)‐5 Months) and GSM4043957 (*Cryba1* cKO‐5 Months). The FASTQ files were analyzed in Partek (v 12.0.0). All pre‐ and post‐alignment QA/QC was performed in Partek. Reads were aligned to whole mm10 genome using STAR (v 2.7.8a). Aligned reads were quantified to mm10 Ensembl Transcripts release 102 using HTSeq (v 0.11.0). Differential gene expression was performed using DESeq2 (v 3.5; Amemiya et al. 2019; Ramirez et al. 2016).

### 4.14 Chromatin Immunoprecipitation (ChIP)

Dissected RPE were incubated with 1% formaldehyde in PBS at room temperature for 10 min. Fixation was stopped by the addition of 1× glycine. TruChIP Chromatin Shearing Kit (Covaris, Cat #: 520127) was used to shear chromatin. Chromatin immunoprecipitation (ChIP) was performed using iDeal ChIP‐seq kit (Diagenode, Cat #: C01010051) on 25 μg of sheared chromatin using 5 μL of H3K27‐Ac antibody (Abcam, Cat #: ab‐4729). Crosslinking was reversed by overnight incubation at 65°C using proteinase K treatment. DNA was then purified using MinElute Reaction Cleanup Kit (Qiagen, Cat #: 28204). To confirm the significant enrichment, qPCR was performed on control genes. Libraries of samples were prepared using a NEBNext Ultra II DNA Library Prep Kit (NEB, Cat #: E7645). The library was sequenced using single‐end reads (150 base pair reads) on a NextSeq 500 system at the ULNV/NIPM Genomics Core.

### 4.15 ChIP‐Seq Data Analysis

FASTQ files from sequencing were aligned to the Mouse mm10 genome in Partek (v 12.0.0) using BWA‐MEM (v 0.7.17). The peaks were identified using MACS2 (v 3.0.0a7), broad region and *q*‐value cutoff of 0.05, compared with respective input samples and annotated using mm10 Ensembl Transcripts release 102. Differential peak analysis was performed comparing cKO H3K27 versus FL H3K27 samples using DESeq2 (v 3.5). Peaks with a *p* value <= 0.05 were considered significant. RET gene acetylation was evaluated by aligning FASTQ files to the reference genome (GRCm38/mm10) using BWA, generating BAM files. Peak calling was performed with MACS to identify regions of significant enrichment, producing BED files. Differential peak analysis was performed using DESeq2 (*p* ≤ 0.05, FC ≥ 1.5) to identify significant acetylation differences between groups. Data were visualized and annotated using the Integrated Genomics Viewer (IGV). The FASQ file for ChIP seq data has been uploaded in Sequence Read Archive (SRA, NCBI) Bio Project ID: PRJNA1142537. To generate TSS plots, BAM files from alignment performed in Partek were downloaded and converted to bigwig files with DeepTools (v 3.5.5) bamCoverage function (Kaarniranta et al. 2023). Reads were extended to a fragment length of 200 base pairs. Read coverage was calculated using a 10‐base pair window and normalized to 1× depth using an effective genome size of 2,650,000,000. Chromosome X was ignored for normalization and blacklisted regions were downloaded from https://github.com/Boyle‐Lab/Blacklist (Amemiya et al. 2019; Ramirez et al. 2016) and excluded. Count matrices for all samples were generated with the DeepTools compute Matrix function using a bin size of 10. TSS were downloaded from the UCSC Table Browser for mm10 NCBI RefSeq Track (https://genome.ucsc.edu/cgi‐bin/hgTables; Amemiya et al. 2019; Ramirez et al. 2016). The reference point was 2000 base pairs above and below TSS. DeepTools plot Heatmap function was used to generate TSS plots. To generate chromosome plots, FL H3K27 samples were averaged with DeepTools bigwigCompare function. The same operation was performed for *Cryba1* cKO H3K27 samples. Integrative Genomics Viewer (v 2.17.4) was used to generate chromosome plot views (https://igv.org/; Amemiya et al. 2019; Ramirez et al. 2016).

### 4.16 Pathway Analysis and Transcription Factor Binding Prediction

Pathway analysis was performed on significant peaks (*p* value <= 0.05) with GREAT v4.0.4 using mouse GRCm38 assembly (http://great.stanford.edu/public/html/). The whole genome was used as the background region. The whole genome was used as the reference set. Transcription factor binding site prediction for MITF in the RET promoter region was performed using the Eukaryotic Promoter Database (EPD) referenced to the mouse genome. Binding sites were identified using a significance threshold of *p* ≤ 0.001 (McLean et al. 2010).

### 4.17 Statistical Analysis

All plots and statistical analyses were performed with Prism 9 (GraphPad) software. Statistical significance was determined by either Student's *t*‐test (two‐tailed) for two groups or 1‐way ANOVA for multiple groups with similar samples. For ER stress protein mediators, western blot analysis was performed, and the data points were tested using the Shapiro–Wilk normality test. For data sets that passed the normality test, statistical analysis was performed using two‐way ANOVA followed by Tukey's post hoc test. Error bars represent the standard deviation of the mean and indicate replicates or the number of animals employed. Results were representative of at least three independent experiments (*n*). Statistical significance was indicated as *p* ≤ 0.05, *p* ≤ 0.01, *p* ≤ 0.001, and *p* ≤ 0.0001.

## Author Contributions

Prasun Guha and D.S. designed the study. S.C. performed histone acetylation studies, HDAC activity‐related experiments, and other biochemical assays, which included endogenous immunoprecipitation studies and inositol rescue experiments. Z.S. and L.V.P. performed NGS data analysis. L.V.P. and N.T. performed biochemical experiments. S.G., V.S.B., S.B., and Pooja Gautam generated, genotyped, and isolated RPE cells for the *Cryba1* ^fl/fl^, *Cryba1* cKO, βA1 KD, and βA3 KO mice. All assays conducted in this research utilized these cells. S.G. conducted the binding assay, V.S.B. helped with the epigenetic and ER stress assays, while S.B. performed the immunofluorescence assay. Additionally, S.G. and S.B. did the RPE explant cultures for in vitro experiments, both with and without specific treatments. E.D. and I.A.D. did the ChIP‐seq assay. Y.S. generated the computer model. M.F.‐B. provided the human tissues and characterized them. S.C. and S.H. designed the figures. K.R. and H.J.J. generated cell‐permeable InsP6. D.S. and Prasun Guha wrote the manuscript. All authors reviewed the results and approved the final version of the manuscript.

## Conflicts of Interest

D.S., S.G., and S.H. have patents on *Cryba1* as a therapy for eye‐related diseases. D.S. is a co‐founder of Ikshana Therapeutics Inc.

## Supporting information


**Figure S1.** *Cryba1* deletion impacts the global transcriptome in mouse RPE cells. Volcano plot (log 10[*p* value] vs. Fold change) displays differentially expressed genes after *Cryba1* deletion. Yellow dots represent upregulated gene expression, whereas green dots represent downregulated gene expression. The y‐axis denotes −log10 *p* value, whereas the x‐axis denotes log2 fold change value. The result is representative of eight individual experiments (*n* = 8).
**Figure S2.** *Cryba1* deletion enhances histone acetylation in mouse RPE cells. Western blot analysis demonstrated that histone acetylation increased in RPE cells from *Cryba1* cKO mice compared to *Cryba1* ^fl/fl^. *Cryba1* ^fl/fl^ RPE cells treated with 2 μM of the pan HDAC inhibitor SAHA was used as a positive control. Immunoblotting was performed against acetylated (A) anti‐H3K14, (B) anti‐H3K9, (C) anti‐H3K18, (D) anti‐H3K27, (E) anti‐H3K56, (F) anti‐H4K5 and (G) anti‐H4K8 (H) anti‐H4K12, and (I) anti‐H4K16 followed by stripping and reprobing against anti‐H3 or anti‐H4 antibodies, respectively, followed by densitometric analysis (J). Results are representative of three individual experiments (*n* = 3).
**Figure S3.** Overexpression of *Cryba1* can rescue HDAC3 activity and H3K9/27 acetylation in RPE cells. (A) p300 activity in *Cryba1* cKO was comparable to *Cryba1* ^fl/fl^. p300 protein was immunoprecipitated from *Cryba1* ^fl/fl^ and *Cryba1* cKO RPE cells, followed by an in vitro histone acetyltransferase (HAT) activity assay. IgG was used as a negative control. Data has been presented as fold change compared with blank value. Immunoblot analysis of p300 from total lysate isolated from *Cryba1* ^*fl/fl*^ and Cryba1 *cKO* RPE cells was represented as input control, while actin was used as loading control. The result was representative of three individual experiments (*n* = 3, NS, not significant) (B) *Cryba1 cKO* RPE cells were stably overexpressed with *Cryba1* plasmid by using an adenoviral transfection procedure (*Cryba1* cKO + *Cryba1*). HDAC3 activity was significantly rescued in *Cryba1* cKO + Ad‐*Cryba1* compared to *Cryba*1^fl/fl^. Lysates isolated from *Cryba1* ^fl/fl^, *Cryba1* cKO, and *Cryba1* cKO + Ad‐*Cryba1* were immunoprecipitated with anti‐HDAC3 antibody followed by an analysis of HDAC3 activity. Immunoblot analysis against anti‐HDAC3 antibody from the lysate isolated from *Cryba1* ^fl/fl^, *Cryba1* cKO, and *Cryba1* cKO + Ad‐*Cryba1* RPE cells was used as control while actin was used as loading control. Results were representative of three independent experiments (*n* = 3, NS, not significant, ****p < 0.0001). (C, D) *Cryba1 cKO* RPE cells were stably overexpressed with wild‐type *Cryba1* adenoviral construct and vector control using an adenoviral transfection procedure. Immunoblot analysis indicated significant rescue in H3K9 and H3K27 acetylation in *Cryba1* cKO + Ad‐*Cryba1*. Lysates isolated from *Cryba1* ^fl/fl^, *Cryba1* cKO, and *Cryba1* cKO + Ad‐*Cryba1* RPE cells were immunoblotted with anti‐H3K9‐Ac (C) or anti‐H3K27‐Ac (D) antibodies. This was followed by stripping and reprobing against anti‐H3 antibodies, which was used as a loading control. Results are representative of three individual experiments (*n* = 3). (E, F) represent densitometric analysis of H3K9/27 acetylation. *n* = 3 (***p < 0.001).
**Figure S4.** Deletion of *Cryba1* in RPE cells influences epigenetic changes. Gene Ontology (GO) enrichment analysis depicting upregulated pathways in *Cryba1 cKO* mouse RPE with enrichment H3K27 acetylation peaks.
**Figure S5.** βA3/A1‐crystallin physically interacts with HDAC3. (A) *In silico* binding analysis predicted *Cryba1* binding with HDAC3 by using 3D computational modeling. βA3/A1‐crystallin (blue), HDAC3 (orange), and SMART‐DAD (green). (B) In the over expression system, βA3/A1‐crystallin‐RFP interacts with endogenous HDAC3. *Cryba1* cKO RPE cells were stably overexpressed with AD‐RFP and βA3/A1‐crystallin‐RFP, respectively, followed by immunoprecipitation with anti‐RFP antibody, then immunoblotted against anti‐HDAC3 antibody. Whole cell lysates from the respective groups were then immunoblotted against anti‐HDAC3 and anti‐RFP antibodies and were represented as input control (*n* = 3). (C) Endogenous βA3‐ and βA1‐crystallin binds to endogenous HDAC3. To analyze endogenous binding between HDAC3, βA3‐ and βA1‐crystallin, cell lysates isolated from *Cryba1* ^*fl/fl*^ and *Cryba1* cKO RPE were immunoprecipitated with anti‐HDAC3 antibody followed by immunoblotting against βA3‐ and βA1‐crystallin. IgG was used as a negative control. Immunoblotting against anti‐HDAC3 and anti‐Cryba1 antibodies from the lysates isolated from *Cryba1* ^fl/fl^ and *Cryba1* cKO was used as input control (*n* = 3). (D) Recombinant βA3‐ and βA1‐crystallin‐myc binds with recombinant HDAC3‐GST in in vitro conditions. βA3‐ and βA1‐crystallin‐myc were incubated with either HDAC3‐GST or GST recombinant protein and then immunoprecipitated with anti‐GST beads followed by immunoblotting against anti‐Myc antibody. Whole cells lysates were immunoblotted against anti‐myc, anti‐HDAC3 and anti‐GST antibodies (input control) (*n* = 3). Results are representative of three individual experiments (*n* = 3). (E) The representative image illustrates the interaction between βA3/A1‐crystallin and HDAC3 within the nucleus of the RPE in flat mounts. The PLA reveals prominent red puncta in the *Cryba1* ^fl/fl^ controls, indicating a robust interaction. Conversely, no puncta are observed in the nucleus of the *Cryba1 cKO* RPE flat mounts, owing to the absence of crystallin. *n* = 3, Scale bar = 10 μm.
**Figure S6.** Overexpression of *Cryba1* can rescue HDAC3 phosphorylation at Ser424 in the *Cryba1* cKO RPE. (A) Adenoviral‐mediated overexpression of *Cryba1* significantly rescued HDAC3 phosphorylation at Serine 424. *Cryba1* cKO RPE cells were stably overexpressed with wild‐type *Cryba1* construct or vector control using an adenoviral transfection procedure. Lysates isolated from *Cryba1* ^fl/fl^, *Cryba1* cKO + vector control, and *Cryba1* cKO + *Cryba1* were immunoblotted with anti‐phospho HDAC3 Ser 424 antibody, followed by stripping and reprobing against an anti‐HDAC3 antibody, which was used as a loading control. (B) Densitometric analysis of pHDAC3 Ser424/HDAC3 acetylation confirms the rescue of HDAC3 phosphorylation at Serine 424. Results are representative of three individual experiments (*n* = 3, ***p < 0.001).
**Figure S7.** Increase in H3K9/H3K27 acetylation in human AMD. (A, B) Immunoblot analysis indicated a significant increase in the level of H3K9 acetylation in AMD patients as compared to healthy human individuals. Lysates isolated from RPE cells of healthy and patients suffering from AMD were immunoblotted with anti‐H3K9‐Ac (A) or anti‐H3K27‐Ac (C, D) antibodies. This was followed by stripping and reprobing against anti‐H3 antibody, which was used as a loading control (*n* = 3). (B, D) represent densitometric analyses of H3K9/27 acetylation. Results are representative of three individual experiments (*n* = 3). *n* = 3 (***p < 0.001).

**Table S1.** Top 10 protein coding genes with increased expression.
**Table S2.** Top 10 protein coding genes with decreased expression.
**Table S3.** Top 10 genes with increased expression and increased acetylation in promoter region (−1 kb to TSS).

## Data Availability

The data that supports the findings of this study are available in the Figures S1–S7 and Tables S1–S3 of this article.
