# Supplementary figures and images for: Age‐Dependent Histone Deacetylase 3 Regulation by βA3/A1‐Crystallin and Inositol Hexaphosphate in Retinal Pigmented Epithelial Cells Reveals a Novel Pathway in Age‐Related Macular Degeneration
Source: Aging Cell. 2025 Jul 15;24(9):e70163. doi: 10.1111/acel.70163 (PMC12419856; doi:10.1111/acel.70163)

**A**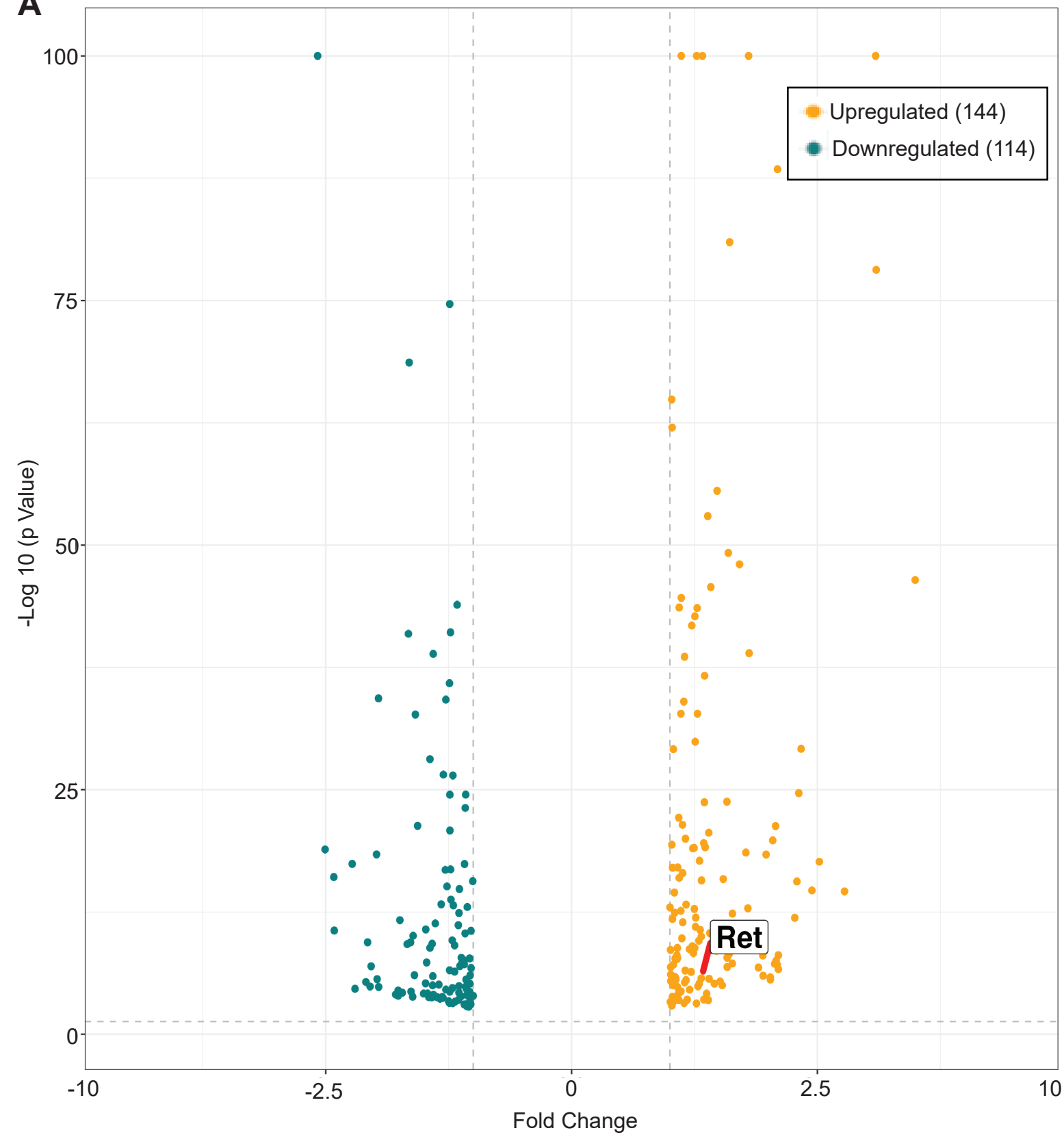

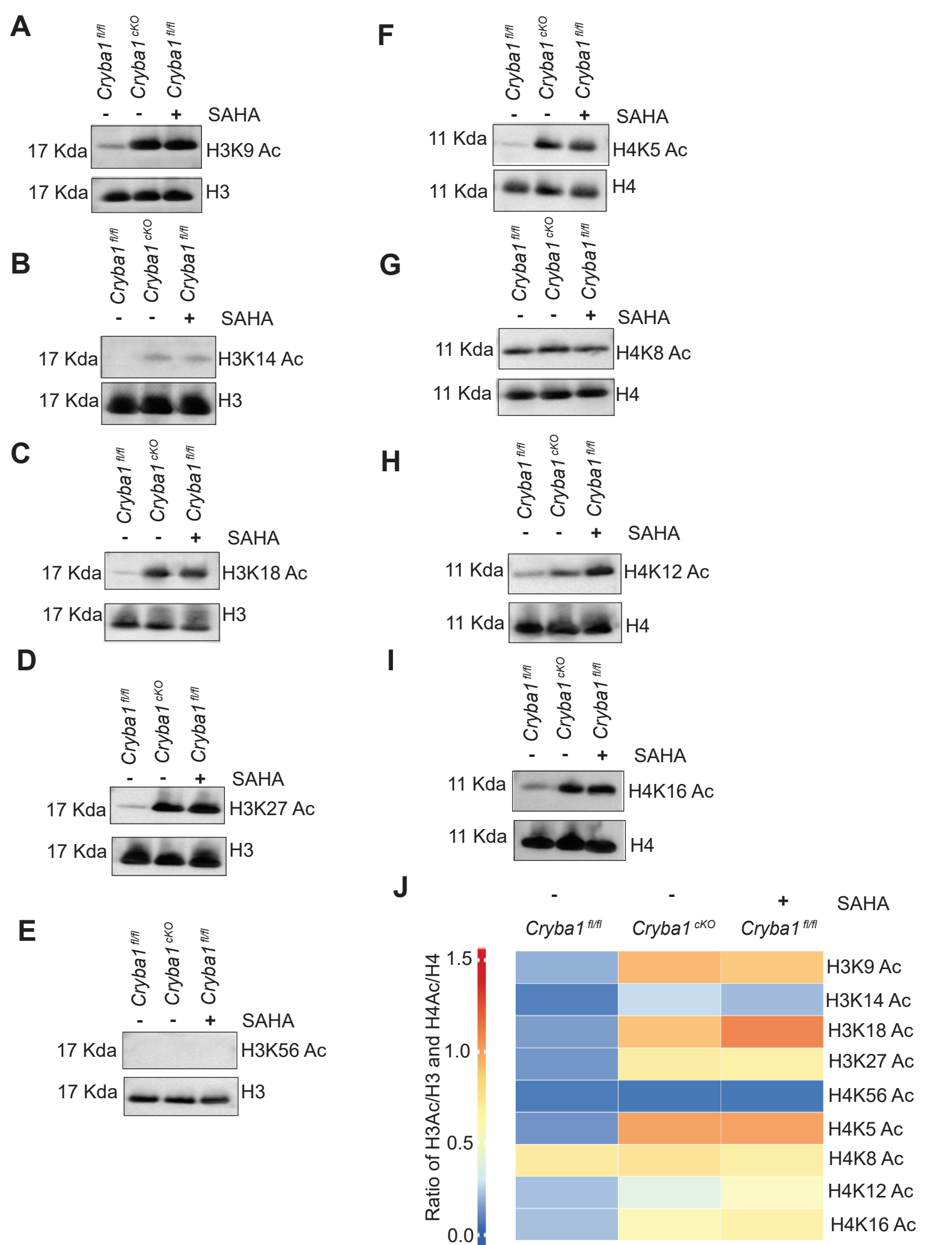

Extended Figure 2



A

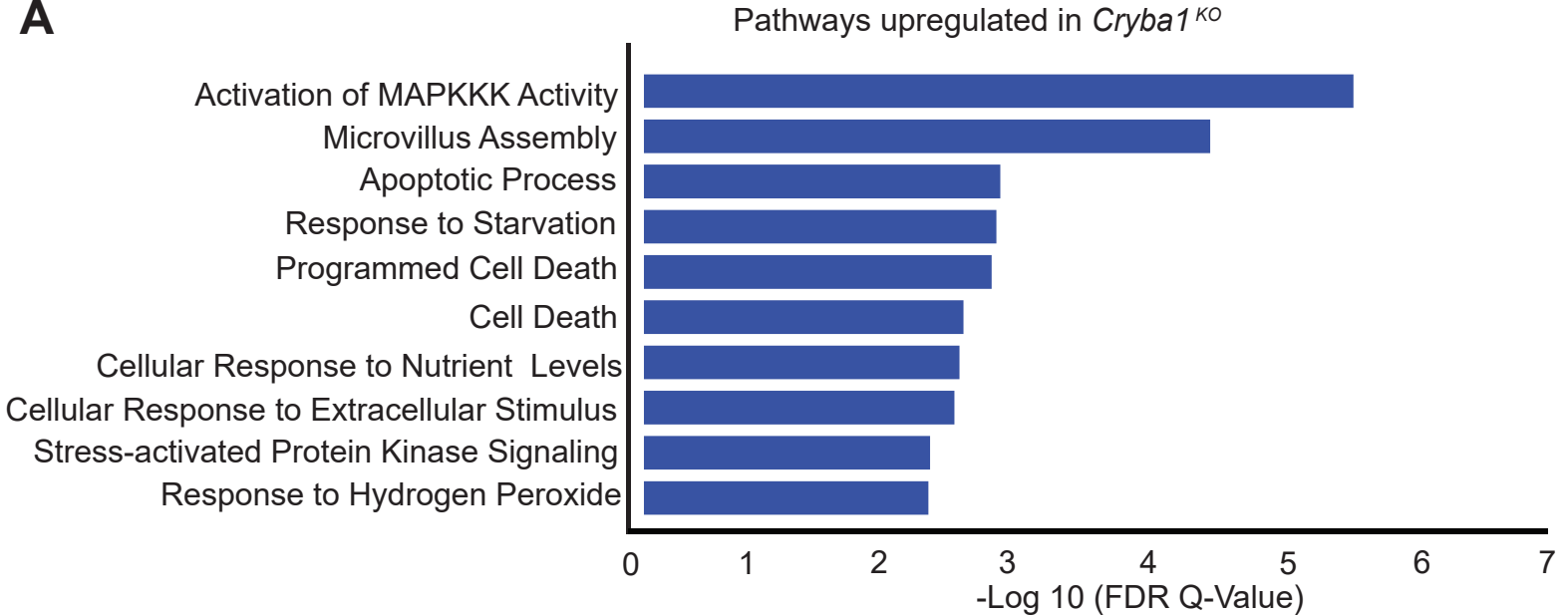

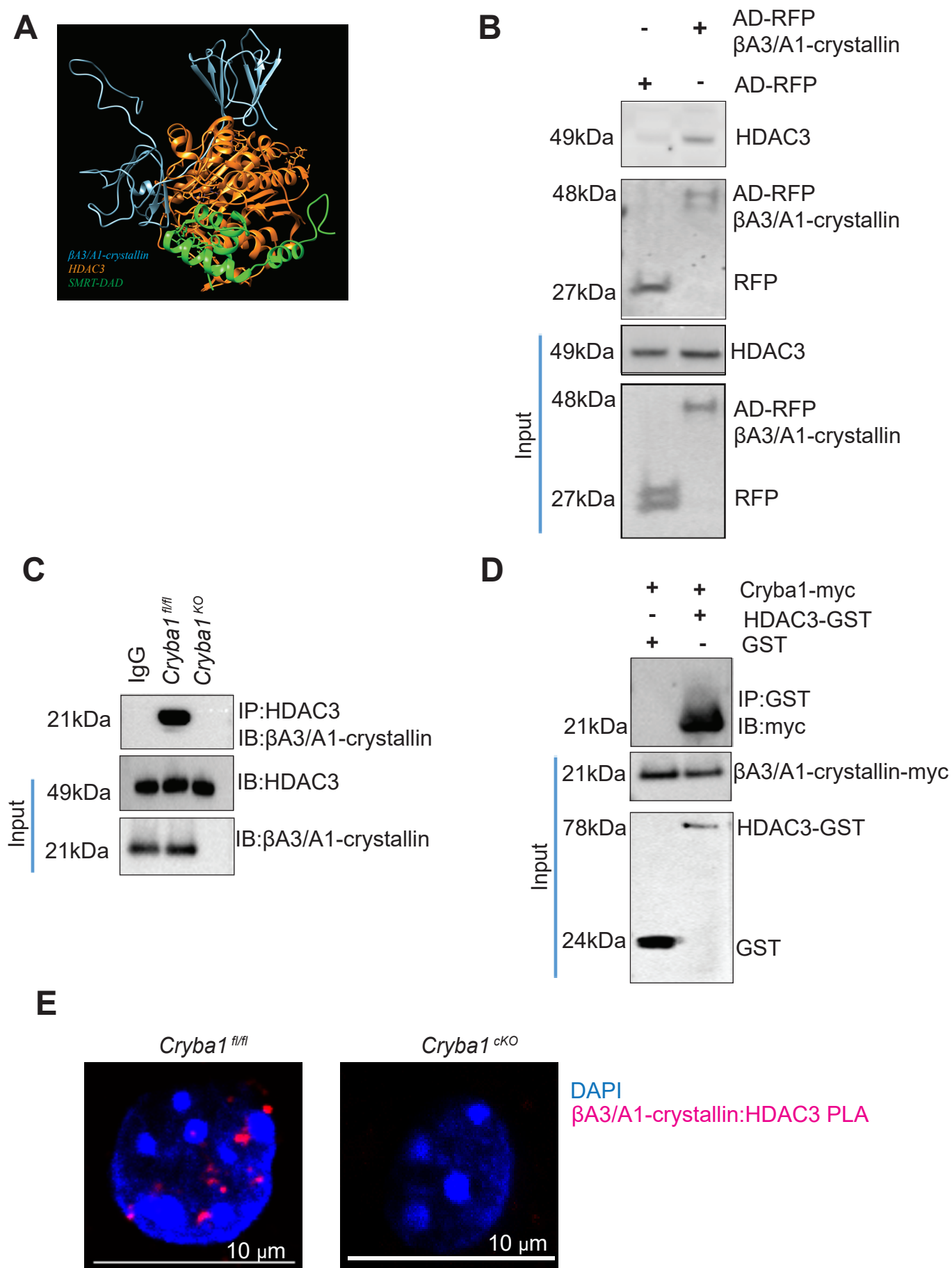

**A**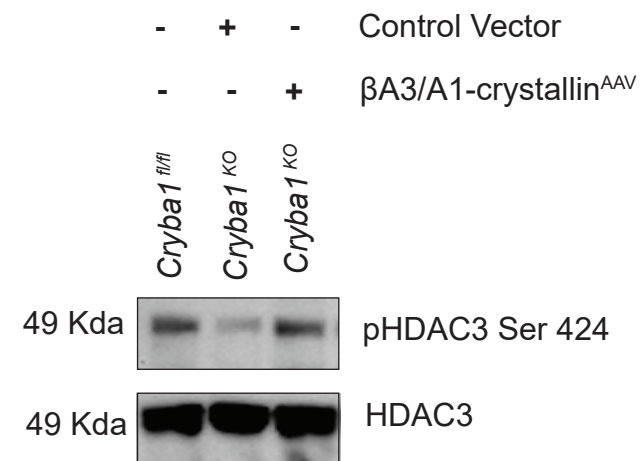**B**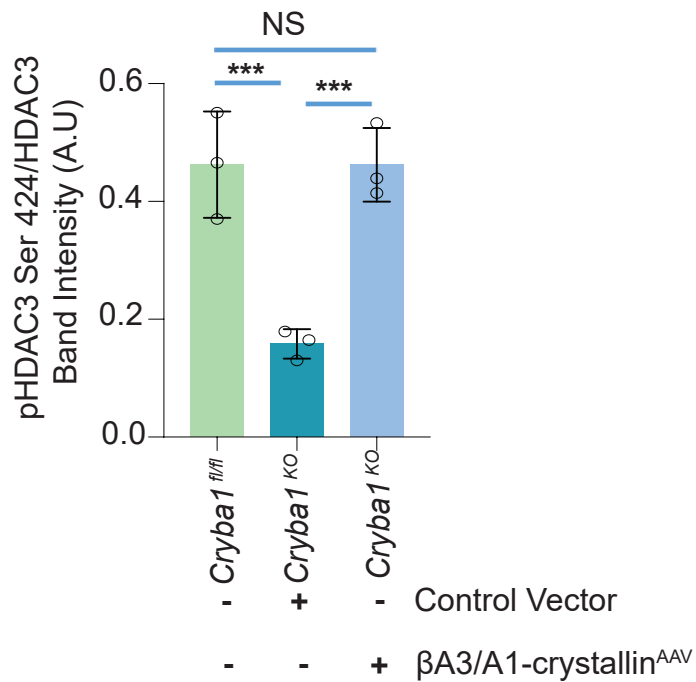

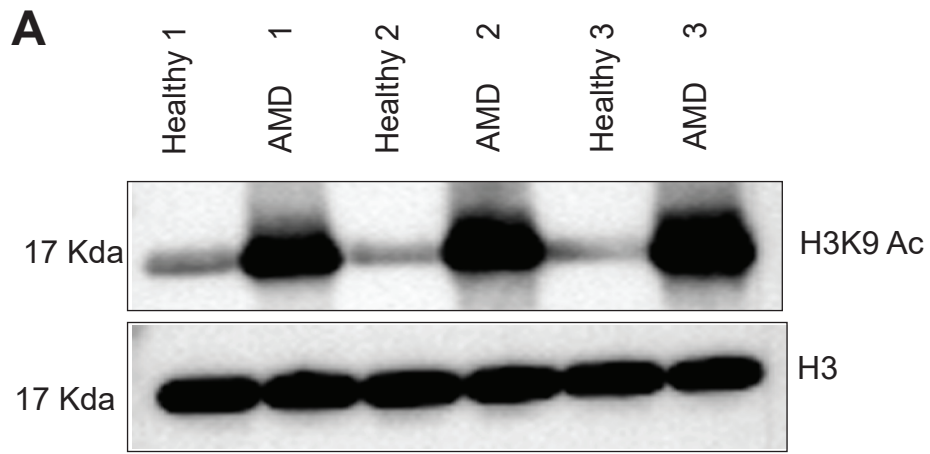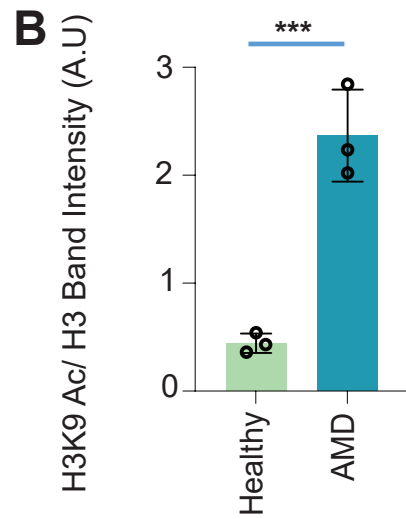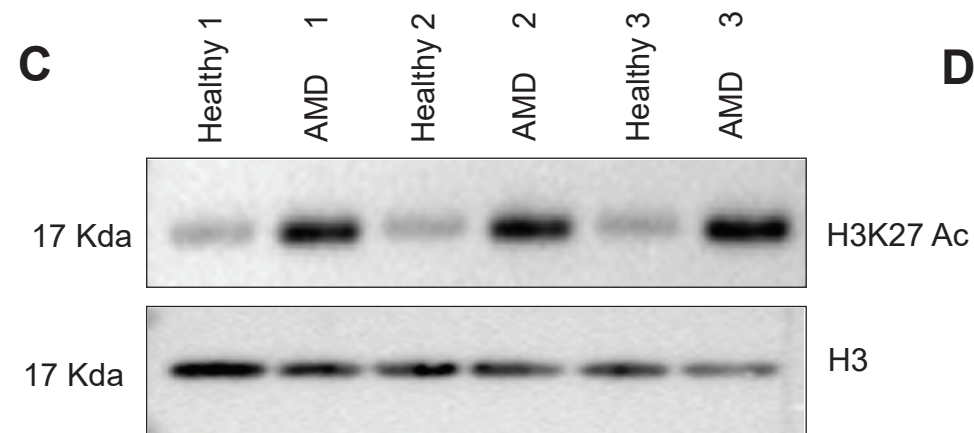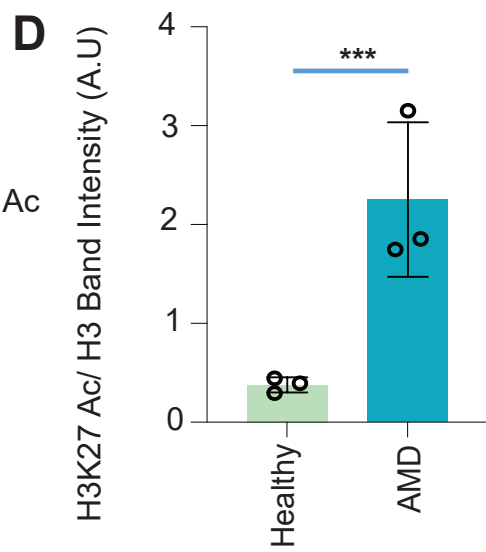

Supplement: Supplementary file 1 — Figure S1. Cryba1 deletion impacts the global transcriptome in mouse RPE cells. Volcano plot (log 10[p value] vs. Fold change) displays differentially expressed genes after Cryba1 deletion. Yellow dots represent upregulated gene expression, whereas green dots represent downregulated gene expression. The y‐axis denotes −log10 p value, whereas the x‐axis denotes log2 fold change value. The result is representative of eight individual experiments (n = 8). Figure S2. Cryba1 deletion enhances histone acetylation in mouse RPE cells. Western blot analysis demonstrated that histone acetylation increased in RPE cells from Cryba1 cKO mice compared to Cryba1 fl/fl. Cryba1 fl/fl RPE cells treated with 2 μM of the pan HDAC inhibitor SAHA was used as a positive control. Immunoblotting was performed against acetylated (A) anti‐H3K14, (B) anti‐H3K9, (C) anti‐H3K18, (D) anti‐H3K27, (E) anti‐H3K56, (F) anti‐H4K5 and (G) anti‐H4K8 (H) anti‐H4K12, and (I) anti‐H4K16 followed by stripping and reprobing against anti‐H3 or anti‐H4 antibodies, respectively, followed by densitometric analysis (J). Results are representative of three individual experiments (n = 3). Figure S3. Overexpression of Cryba1 can rescue HDAC3 activity and H3K9/27 acetylation in RPE cells. (A) p300 activity in Cryba1 cKO was comparable to Cryba1 fl/fl. p300 protein was immunoprecipitated from Cryba1 fl/fl and Cryba1 cKO RPE cells, followed by an in vitro histone acetyltransferase (HAT) activity assay. IgG was used as a negative control. Data has been presented as fold change compared with blank value. Immunoblot analysis of p300 from total lysate isolated from Cryba1 fl/fl and Cryba1 cKO RPE cells was represented as input control, while actin was used as loading control. The result was representative of three individual experiments (n = 3, NS, not significant) (B) Cryba1 cKO RPE cells were stably overexpressed with Cryba1 plasmid by using an adenoviral transfection procedure (Cryba1 cKO + Cryba1). HDAC3 activity was s [file ACEL-24-e70163-s001.pdf]
