# Supplementary material for: Age‐Dependent Histone Deacetylase 3 Regulation by βA3/A1‐Crystallin and Inositol Hexaphosphate in Retinal Pigmented Epithelial Cells Reveals a Novel Pathway in Age‐Related Macular Degeneration
Source: Aging Cell. 2025 Jul 15;24(9):e70163. doi: 10.1111/acel.70163 (PMC12419856; doi:10.1111/acel.70163)
Supplement: Supplementary file 2 — Table S1. Top 10 protein coding genes with increased expression. Table S2. Top 10 protein coding genes with decreased expression. Table S3. Top 10 genes with increased expression and increased acetylation in promoter region (−1 kb to TSS). [file ACEL-24-e70163-s002.pdf]

**Table 1.** Top 10 protein-coding genes with increased expression.

| | Fold Change Expr. | Description |
|---|---|---|
| Muc4 | 18.66 | mucin 4 |
| Grin3a | 8.97 | glutamate receptor ionotropic, NMDA3A |
| Egr1 | 8.65 | early growth response 1 |
| Ifit1bl1 | 6.71 | interferon induced protein with tetratricpeptide repeats 1B like 1 |
| Mmp9 | 6.52 | matrix metallopeptidase 9 |
| Fos | 5.78 | FBJ osteosarcoma oncogene |
| Egr3 | 5.62 | early growth response 3 |
| Asprv1 | 5.41 | aspartic peptidase, retroviral-like 1 |
| Ptgs2 | 5.35 | prostaglandin-endoperoxide synthase 2 |
| Trim30d | 5.21 | tripartite motif-containing 30D |

**Table 2.** Top 10 protein-coding genes with decreased expression.

| Gene name | Fold Change Expr. | Description |
|---|---|---|
| Reln | -20.6811 | reelin |
| Pax6 | -8.12821 | paired box 6 |
| Acta1 | -7.30407 | actin alpha 1, skeletal muscle |
| Dipk1c | -6.58863 | divergent protein kinase domain 1C |
| Mmp3 | -6.11063 | matrix metallopeptidase 3 |
| Tgm1 | -5.77113 | transglutaminase 1, K polypeptide |
| Tnni2 | -5.59096 | troponin I, skeletal, fast 2 |
| Mylpf | -5.58699 | myosin light chain, phosphorylatable, fast skeletal muscle |
| Sfrp4 | -5.15246 | secreted frizzled-related protein 4 |
| Slc13a4 | -5.10024 | solute carrier family 13 (sodium/sulfate symporters), member 4 |

**Table 3.** Top 10 genes with increased expression and increased acetylation in promoter region (-1kb to TSS)

| Gene name | Fold Change Expr. | Fold Change Promoter Ac. | Description |
|---|---|---|---|
| Ret | 2.95 | 1.88 | ret proto-oncogene |
| Tmem30b | 1.86 | 2.95 | transmembrane protein 30B |
| Elk4 | 1.25 | 2.74 | ELK4, member of ETS oncogene family |
| Lrp2 | 1.33 | 2.48 | low density lipoprotein receptor-related protein 2 |
| Tent4b | 1.26 | 2.39 | terminal nucleotidyltransferase 4B |
| Prdm11 | 1.30 | 2.13 | PR domain containing 11 |
| Rab11fip4 | 1.40 | 2.10 | RAB11 family interacting protein 4 (class II) |
| Cdh23 | 2.59 | 1.78 | cadherin related 23 (otocadherin) |
| Cldn10 | 3.18 | 1.75 | claudin 10 |
| Oasl1 | 2.13 | 1.63 | 2'-5' oligoadenylate synthetase-like 1 |
